# Human occupations of upland and cold environments in inland Spain during the Last Glacial Maximum and Heinrich Stadial 1: The new Magdalenian sequence of Charco Verde II

**DOI:** 10.1371/journal.pone.0291516

**Published:** 2023-10-04

**Authors:** Javier Aragoncillo-del Río, José-Javier Alcolea-González, Luis Luque, Samuel Castillo-Jiménez, Guillermo Jiménez-Gisbert, José-Antonio López-Sáez, José-Manuel Maíllo-Fernández, Mónica Ruiz-Alonso, Ignacio Triguero, José Yravedra, Manuel Alcaraz-Castaño

**Affiliations:** 1 Molina-Alto Tajo UNESCO Global Geopark, Molina de Aragón, Guadalajara, Spain; 2 Escuela Internacional de Doctorado de la UNED (EIDUNED), Madrid, Spain; 3 Area of Prehistory (Department of History and Philosophy), University of Alcalá, Alcalá de Henares, Spain; 4 Environmental Archeology Research Group, Institute of History, CCHS CSIC, Madrid, Spain; 5 Institute of Evolution in Africa (IDEA), University of Alcalá, Madrid, Spain; 6 Department of Prehistory and Archaeology, Universidad Nacional de Educación a Distancia (UNED), Madrid, Spain; 7 Department of Prehistory, Ancient History and Archaeology, Complutense University, Madrid, Spain; Universita degli Studi di Milano, ITALY

## Abstract

The settlement of cold and arid environments by Pleistocene hunter-gatherers has been a heated topic in Paleolithic Archaeology and the Quaternary Sciences for years. In the Iberian Peninsula, a key area for studying human adaptations to such environments is composed by the large interior and upland regions of the northern and southern plateaus (*Mesetas*) and bordering areas. As, traditionally, these regions have been relatively under-investigated compared to the ecologically more favored coastal areas of the peninsula, our knowledge of the human settlement of the whole Iberian hinterland remains scarce for the Last Glacial. In this paper we present the discovery and first geoarcheological, paleoenvironmental and chronometric evidence obtained at Charco Verde II, a new site close to the southwestern foothills of the Iberian system range (Guadalajara province, Spain), bearing a sequence of Magdalenian human occupations starting at least at 20.8–21.4 ka cal BP during the Last Glacial Maximum, and covering Greenland Stadial 2 until ∼15.1–16.6 ka cal BP, including Heinrich stadial 1. As this site is located in an upland region which today faces one of the harshest climates in Iberia, such occupation sequence, occurred during some of the coldest and most arid phases of the Last Glacial, has relevant implications for our understanding of human-environment-climate interactions and population dynamics in Iberia and Western Europe. These findings support the hypothesis that the Iberian hinterland was not avoided by Upper Paleolithic hunter-gatherers due to ecological constraints, but it hosted a complex and relatively dense settlement at least in some areas, even during cold periods. This suggest, one more time, that the historical scarcity of Upper Paleolithic sites in inland Iberia is, to a significant extent, an artifact of research bias.

## Introduction

### Population dynamics in inland Iberia during the Last Glacial and the discovery of the Charco Verde II archeological sequence

The Iberian Peninsula, including modern-day Spain and Portugal, has been a hotspot for our knowledge of the Upper Paleolithic human societies since the beginnings of the 20^th^ century [1–5]. Long-considered part of a southwest European refugium during the coldest periods of the last glacial cycle (∼115.0–11.7 ka cal BP) [6–9], Iberia is thought to have been home for people and animals moving from the northern latitudes of Europe during the Last Glacial (Marine Isotope Stages 4, 3 and 2–73.5–11.7 ka cal BP), and especially during its harshest peaks [10], such as the Last Glacial Maximum (LGM) (26.5–19 ka cal BP *sensu* [11]) or the Heinrich stadials (HS) [12]. However, given that most of the Upper Paleolithic archeological and paleoanthropological record of Iberia has been historically concentrated, and often limited, to the coastal areas of the peninsula, the large hinterland in-between the Cantabrian, Mediterranean and Atlantic regions, has been considered nearly irrelevant for our understanding of the Upper Paleolithic settlement of Southwest Europe [13–16]. Therefore, there is a marked contrast between the heavily populated Iberian coastal regions on the one hand, and the Iberian interior, often regarded as a virtually “no-man’s” land–especially for its the early and middle phases of the Upper Paleolithic–on the other [17].

**Fig 1:**
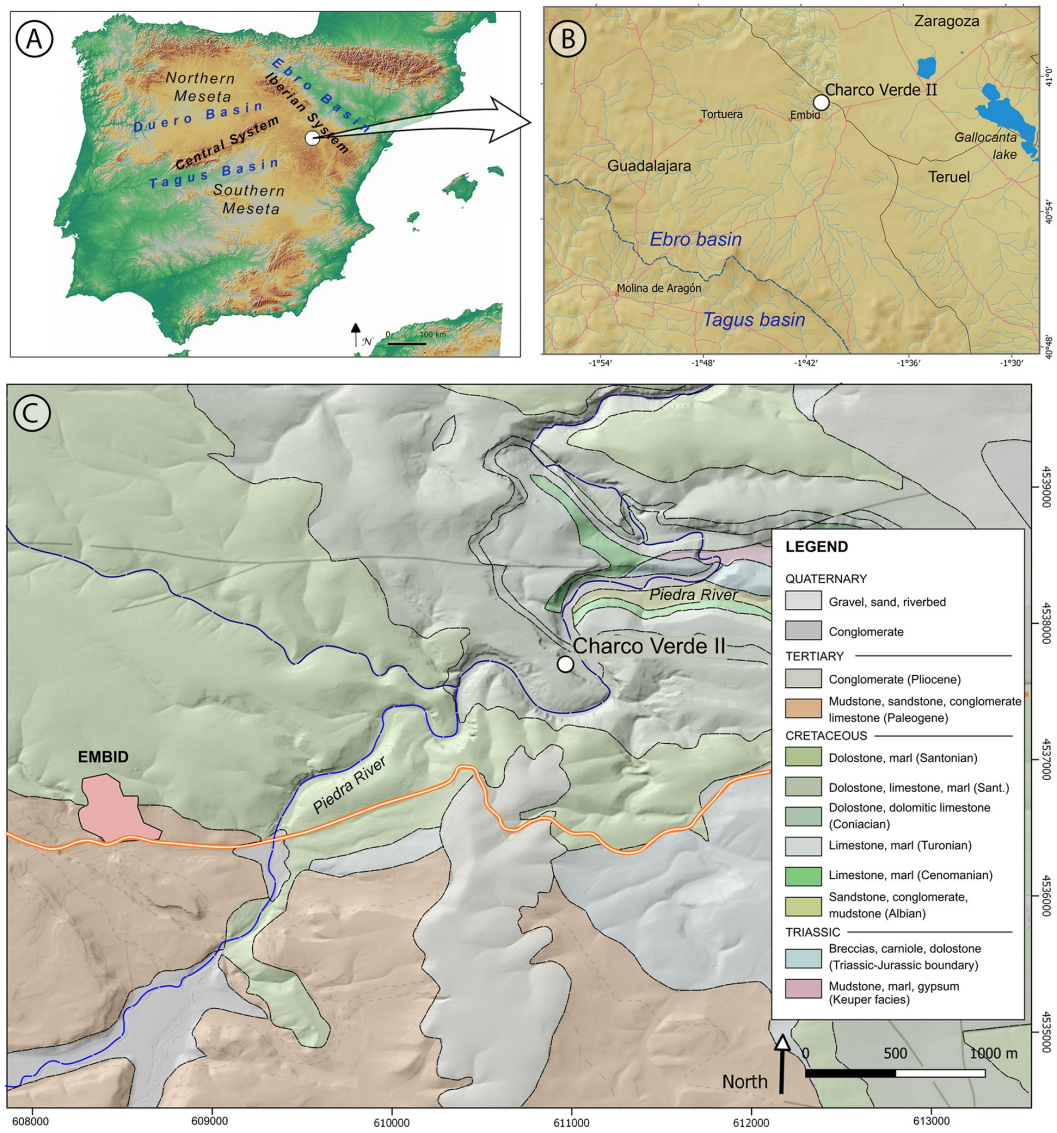
Location of the Charco Verde II rock shelter in the Iberian Peninsula. (A), in the Geographic and relief map of the study area (B) and in the Geological map of the study area (C). Base cartographic data in (A) and (B) obtained from Centro Nacional de Información Geográfica, modified from PNOA_MDT200_ETRS89_HU30 csn.es (free for use: https://centrodedescargas.cnig.es/CentroDescargas/index.jsp). Base cartographic data in (C) obtained from Spanish Geological Survey, IGME, GEODE. Mapa Geológico Digital continuo de España [online] [07/03/2022] (free for use: http://info.igme.es/cartografiadigital/geologica/geodezona.aspx?Id=Z1700).

The inland territories of Iberia are composed of a large upland plateau (the *Meseta*) divided into the Northern and Southern *Mesetas* by the Central System Mountain range (Fig 1). Considered as a whole, the relatively high altitude and the continentalized Mediterranean climate of inland Iberia (and especially of the Northern Meseta and the north-eastern part of the Southern one, dominated by the Supramediterranean biogeographical region), may represent a potentially unsuitable landscape as compared to those of the lower and more temperate regions of the Iberian coasts. Both classic archeological studies and recent modeling works on Late Pleistocene habitat suitability have stressed this potentially risky nature of inland Iberia for the settlement of hunter-gatherers during the Last Glacial. These studies usually limit the presence of humans in the interior to short-term or sporadic incursions during warm intervals, and only recognize an effective settlement of central Iberia once the Last Glacial Maximum finished around 19000 years ago [13, 18–22]. Factors specifically pointed as main drivers of the supposed lack of settlement of these inland regions do not only revolve around high altitudes, cold climates and harsh environments [23, 24], but also stress high climate variability [25], risk of resource failure [26] and low values of ecosystem productivity [27]. However, the increasing refinement of these models and, more prominently, recent results due to increasing efforts on archeological field surveys and excavations, allow moving forward on discussing Last Glacial population dynamics in inland Iberia based on something more than mathematical modeling and the biogeographic features of its landscapes.

Today, recent field data has strengthened the hypotheses that (1) the Last Glacial human settlement of at least some regions of inland Iberia was more recurrent and complex than previously thought, and (2) that this settlement, while influenced by climatic and environmental factors, was not impeded by them. There is an increasing number of Solutrean occurrences, including rock art sites, found at different inland regions [28]. The most relevant are the Manzanares valley in the Madrid basin [29], the southeastern foothills of the Central System range [30–32], and the area of the Duero basin bordering Spain and Portugal [33–35]. Gravettian settlement is also found in the Portuguese Douro Basin [34–36], as well as in central Iberia, albeit still limited to the most recent phases of the technocomplex [17, 28, 31]. Finally, to date, the Aurignacian has only been clearly defined in the single site of Cardina-Salto do Boi in the Côa River valley [34], and only for its Evolved phase, while this cultural tradition remains elusive in the rest of inland Iberia (but see [17, 28]). Overall, although there is still an important gap between the last Neanderthal presence in inland Iberia, currently dated to ∼42 ka cal BP in the central areas [37, 38] and to a slightly younger age at Cardina-Salto do Boi [34], and the first modern human occupation of the Iberian hinterland, the big picture is clearly moving on. There is increasing evidence supporting the idea that the depiction of inland Iberia as either a desolate landscape or a mere crossing-area during most of the Upper Paleolithic, was a biased picture derived from the preference of researchers towards the coastal regions of the peninsula, to the expense of the large Iberian hinterland. In fact, this has been pointed out by many researchers during the last years [14–16, 20, 29, 30, 34, 35, 39–47]. Furthermore, and more significantly, some of the human occupations recently recorded in inland Iberia occurred precisely during the coldest peaks of the Last Glacial, such as HS 2, and hence call into question the idea that humans avoided the Iberian interior due to environmental and climatic constraints. This is the case of the pre-Solutrean occupations documented at the Peña Capón site, which demonstrate the recurrent presence of hunter-gatherer groups in the southeastern foothills of the Central System range in a context of harsh climatic and environmental conditions [32]. It is true that some heavily investigated regions, such as the Atapuerca area [48, 49], or some parts of the Middle Duero basin [50] have shown no significant evidence of Upper Paleolithic settlement, while yielding a dense record of Lower and Middle Paleolithic occupations–which, nonetheless, mostly corresponds to the Middle Pleistocene. However, this trend cannot be translated to the whole Iberian hinterland, where a large portion of the territory remains unexplored. Specific research projects aimed at the location of Late Pleistocene open-air deposits (mostly lower fluvial terraces), which are a potentially relevant loci for the Upper Paleolithic settlement in inland Iberia, have been few to date [17, 34, 51, 52]. Our hypothesis does not assume that once more research is developed in inland Iberia the emerging picture on patterns of settlement and land use will be similar to that recorded in the Cantabrian, Mediterranean or Atlantic regions, as there is strong evidence that this will not be the case. Our point is that, in order to properly understand population dynamics and human-environment-climate interactions in inland Iberia, specific projects aimed at these issues have been historically few, and hence need to be reinforced to test competing hypotheses.

**Fig 2:**
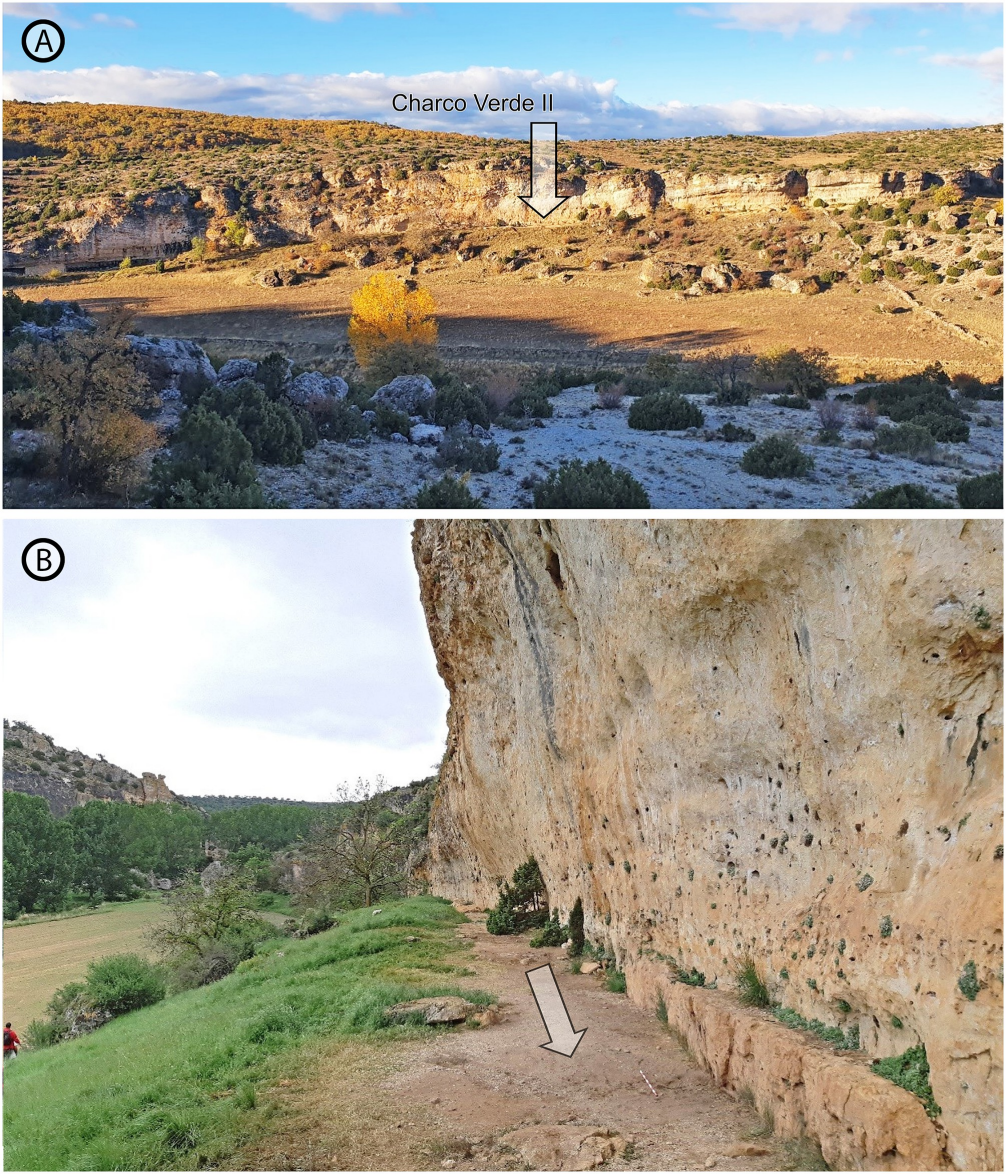
A) Panoramic view of the dolostone escarpment in the Piedra River valley, showing the location of Charco Verde II (white arrow points to the excavation area). B) View from the West of the sedimentary deposit preserved at the foot of the rock shelter.

**Fig 3:**
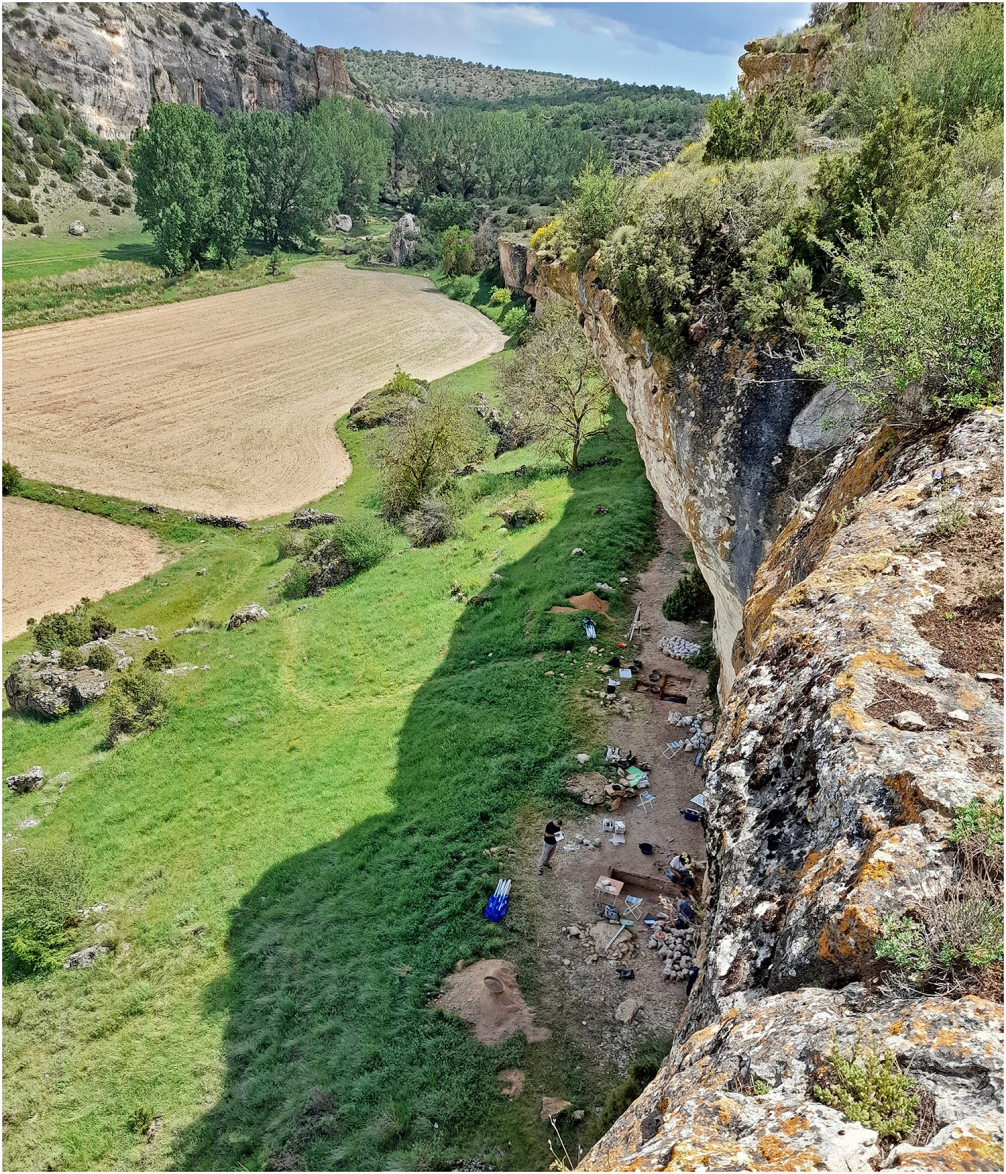
View from the top of the Charco Verde II archeological deposit during the 2021 excavation season.

Within this research context, our team has been working during the last years under the general hypothesis that the human settlement of inland Iberia was more abundant and complex than previously believed, both during the last stages of the Middle Paleolithic and the Upper Paleolithic [16, 17, 29–32, 37, 45, 53]. To test this idea, the systematic and geoarcheological field survey of different regions of the Iberian hinterland was a much-needed task. We started this task in 2018 and, among the areas surveyed, the eastern part of the Guadalajara province (Spain), close to the southwestern foothills of the Iberian System range, has been a relevant locus. This area is dominated by the karst environments and valleys of the Molina moorlands and the *Alto Tajo* region (Upper Tagus basin) (Fig 1), where previous surveys had been conducted by two of us (J. Aragoncillo and J.M. Maíllo) since 2017. The archeological finds recorded at different locations of the Piedra and Mesa River valleys, together with the sedimentary features of their deposits, led the whole team to carry out intensive surveys in some of these locations during 2019. Among them, the karstic formations of the Piedra River valley, and namely the so-called Charco Verde II locality, exhibited a great potential. This site is located under a dolostone rock shelter, very close to the river course, in the municipality of Embid (Guadalajara province) (Figs 1B, 1C, 2 and 3). During the field surveys we found Paleolithic-like lithic assemblages in the surroundings of these dolostone outcrops, including a dihedral burin on flint (Fig 13) and several blade blanks right at the foot of the cliff. Together with the preliminary observations on the sedimentary deposit where these artifacts were recorded, which showed no significant post-depositional disturbance (Figs 2 and 3), these findings led us to conduct a systematic excavation at the site.

In this paper we report the first results obtained after two field seasons at Charco Verde II, including geological and geomorphological descriptions, recording of the stratigraphic sequence, archaeozoological and archaeobotanical data (macromammals, pollen and wood charcoal), a techno-typological characterization of the archeological assemblages focused on Level 1, and first radiocarbon dates. Although research at the site will follow up in the coming years, data obtained thus far have shown a significant impact for our understanding of the Last Glacial settlement of inland Iberia, especially during MIS 2.

### Geographical and geological context

**Fig 4:**
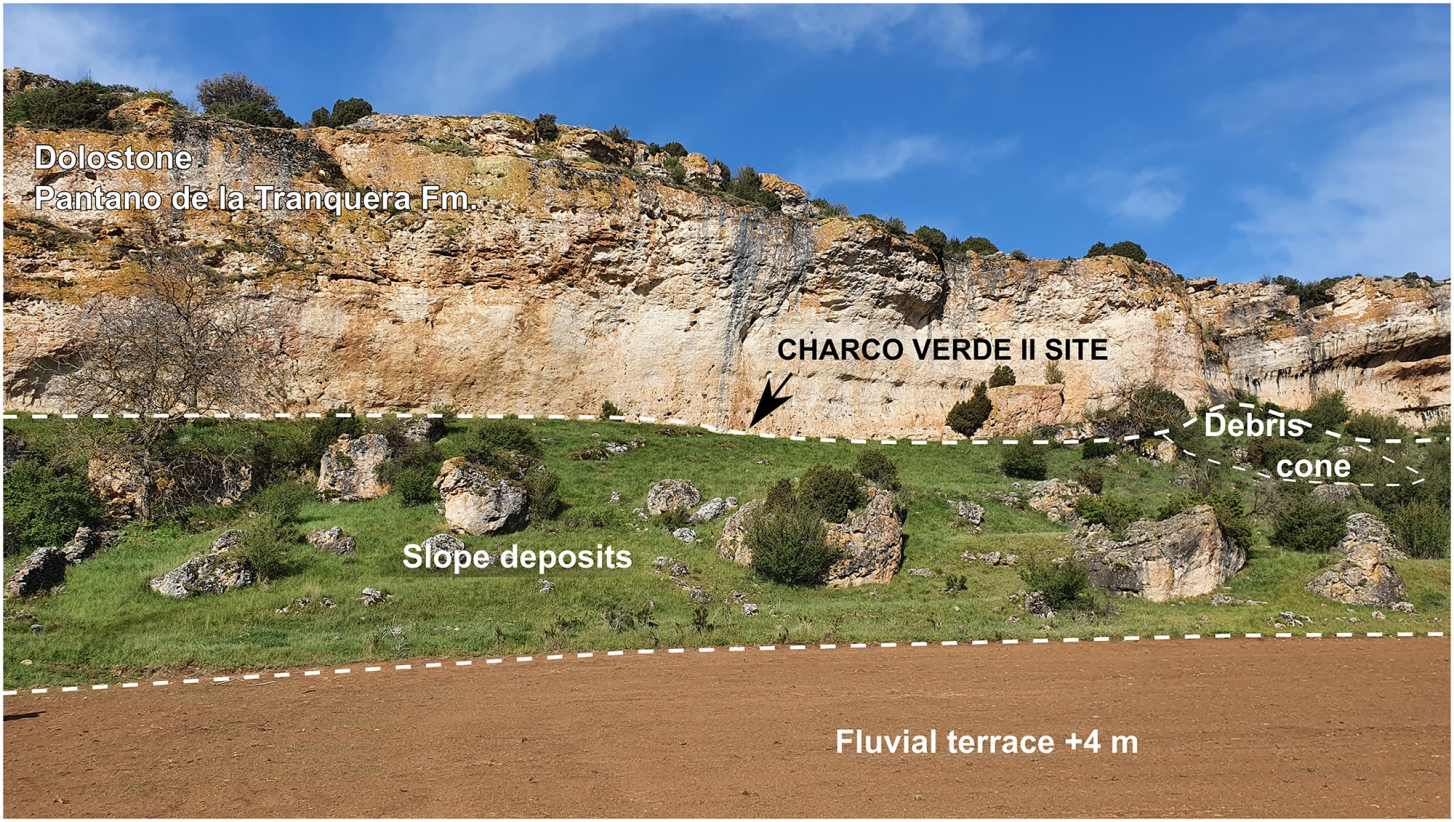
Cretaceous stratigraphic units and Quaternary deposits outcropping in the Charco Verde II site area.

The Charco Verde II site is located at an altitude of 1050 m above mean sea level (amsl), at the foot of a limestone-dolostone escarpment belonging to the Cretaceous Pantano de la Tranquera Formation (Coniacian–Upper Santonian) (Fig 1C), and at a relative elevation of +25 m above the current watercourse of the Piedra River. The deposits bearing archeological remains are extended on a narrow and elongated platform–with an approximate average width of 5 m and a longitude of almost 150 m–which consists of loose, poorly sorted, yellowish-beige in colour carbonate silty sands, mixed with large angular dolomite rock fragments fallen from the rock wall and slope. It is located 21 m above the most recent fluvial terrace and flood plain recorded in the area, +2–4 m above the narrow channel of the river (Fig 4). The deposit gently slopes upstream and seems to extend homogeneously, almost connecting with the fluvial terrace level. We do not yet know if the archeological deposit is continuous in this whole extent.

The situation of the site is related to the Piedra River headwaters in the Tortuera-La Yunta depression, at the foothills of the Sierra de Caldereros, relief that divides the Tagus and Ebro basins in this area. The sediments bearing the archeological remains were accumulated as a colluvium at the foot of a 16-meter-high escarpment of the above mentioned massive Late Cretaceous dolostone and dolomitic limestone located in the left bank of the river (Fig 2). This substrate of Mesozoic carbonate rocks is part of an elongated calcareous outcrop, oriented from northwest to southeast, located in the central sector of the Iberian System Mountain range (Fig 4) [54]. The relief of the area consists of gently sloping hills, with a height between 1,000 and 1,200 m amsl. Its morphology is mainly conditioned by both tectonics and three erosional surfaces developed at the end of the Tertiary and during the Quaternary [55]. The most abrupt reliefs are due to the incision of the Quaternary fluvial network on these carbonate rocks during the Middle or Late Pleistocene [56]. The karstification of the limestone and dolostone is not very intense near the site, despite having started at the beginning of the Pliocene, and despite the fact that the most massive dolomitic units of the upper Cretaceous are favorable facies for caves formation. However, large exokarstic forms are found nearby, such as some dolines and karstic pools located few kilometers away, or the polje that gave rise to the close Gallocanta lake [56, 57]. A few small caves are also found nearby, some of them bearing Quaternary endokarstic deposits, yet no archeological or paleontological evidence has been identified in them thus far.

**Fig 5:**
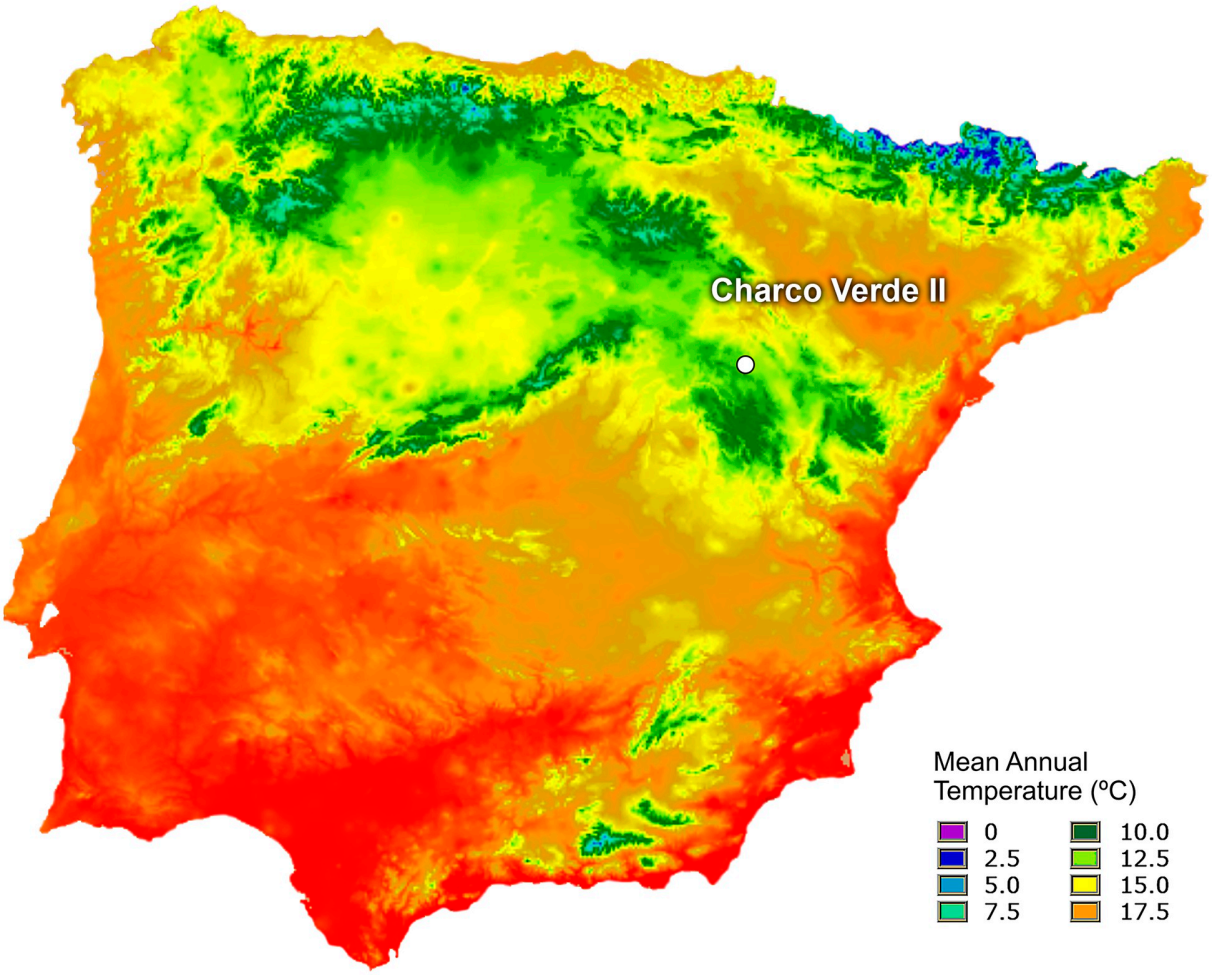
Location of Charco Verde II in the Iberian Peninsula and current mean annual temperatures. Base cartographic data obtained from Atlas climático digital de la Península Ibérica, Universidad Autónoma de Barcelona *(UAB)* (free for use: https://opengis.grumets.cat/wms/iberia/espanol/es_cartografia.htm).

As mentioned above, Charco Verde II is located in a region which nowadays faces one of the most extreme climates in the Iberian Peninsula, with an annual mean temperature of 9,6 ºC [58, 59] (Fig 5) and having reached -20 ºC during several winters in the second half of the 20^th^ century [60]. In fact, the nearby village of Molina de Aragón (20 km away from the site) holds one of the records of minimum temperatures reached to date for populated regions in Spain, with -28,2 ºC [60].

## Objectives and methods

### Permits and repositories

All necessary permits were obtained for the described study, which complied with all relevant regulations. Fieldworks at Charco Verde II were authorized by the *Dirección General de Cultura de la Junta de Comunidades de Castilla–La Mancha* (Spain) (Exp.: 20.1677 and Exp.: 21.1374) with permission from the landowners, Javier Vidal and family.

Archeological assemblages from Charco Verde II are housed in the History and Philosophy Department (Prehistory Area) of the University of Alcalá and the *Museo de Guadalajara* (Guadalajara, Spain). Both repositories are accessible for all researchers upon request.

### Fieldwork: Excavation, recording and sampling

The main objective leading us to conduct a systematic excavation at Charco Verde II was finding new evidence for testing our hypothesis on the population dynamics and human-climate-environment interactions in inland Iberia during the Last Glacial as synthetized above [see also 17]. To do so, bringing into light new archeological and paleoenvironmental data related to the coldest stages of the last glaciation, such as the Last Glacial Maximum or the Heinrich stadials, were considered potentially high-impact results. Furthermore, given the geographic location of Charco Verde II, at the drainage divide between the Ebro and Tagus basins, and especially its altitude of 1050 m above mean sea level and its harsh climatic and environmental setting, added even more relevance to our potential findings. In other words, documenting human presence during the coldest phases of the last glaciation in a geographic region which nowadays faces one of the harshest climates and environments in Iberia (see below), would be rock-solid evidence supporting the hypothesis that Upper Paleolithic hunter-gatherers settled the upland regions of inland Iberia even during severe environmental conditions. Yet, the lithic assemblage recovered in the field surveys, relatively scarce and undiagnostic despite the presence of a burin, only suggested an Upper Paleolithic chronology for the potential site to be discovered, being more accurate chrono-cultural assignments still unwarranted.

**Fig 6:**
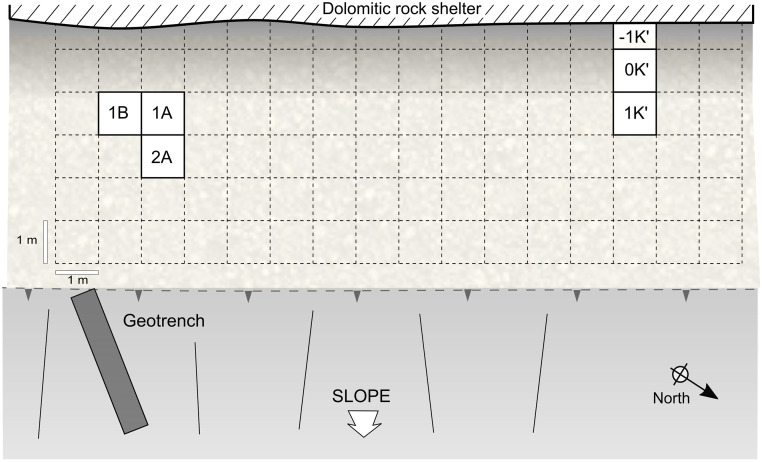
Schematic plan of the archeological excavation in relation to the rock shelter’s wall.

**Fig 7:**
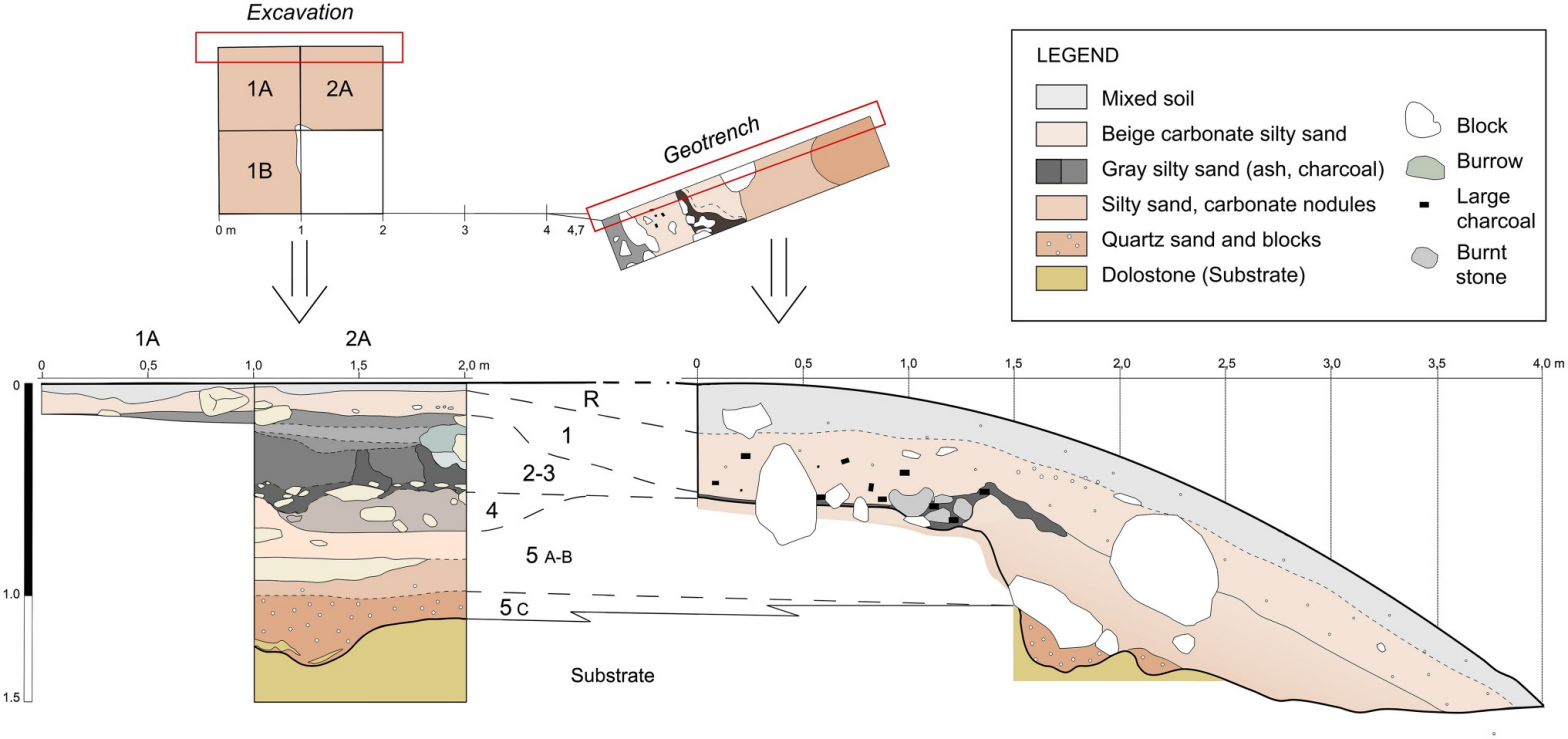
Stratigraphic sequences recorded in the test pit of Square 1A (left) and the Geotrench (right).

To date, we have conducted two excavation seasons at Charco Verde II, in the Autumn of 2020 and the Spring of 2021. The field objectives during these two seasons were (1) recording archeological evidence of the potential Upper Paleolithic human presence preserved at the site, (2) date such a presence, and (3) know the spatial extension and stratigraphic sequence of the archeological deposit as a first step to investigate the function, nature, and chronological depth of the settlement at the site. In the first season we started the excavation of three square meters in a central area of the deposit, very close to the place where we found the burin the year before. This excavation was complemented with a ‘Geotrench’ conducted at the slope of the deposit, with the aim of documenting the stratigraphic sequence as soon and as much as possible. In the second field season (2021) we continued excavating the three square meters in the central area of the site, albeit we transformed one of them into a test pit, with the aim of quickly recording the whole stratigraphic sequence at this central area. Furthermore, 10 meters to the northwest we opened another 2,5 square meters, in order to delimitate the horizonal extension of the archeological site (Figs 3 and 6). Each square meter to be excavated was spatially located according to a grid based on a relative system of cartesian coordinates, for which the E-W axis was aligned with the rock shelter’s wall. Squares on this axis were named after letters, while for the perpendicular axis we used numbers (Fig 6). Thus, in squares 1B, 2A, 0K’ and 1K’ we reached a depth of ∼20 cm, while in the test pit of square 1A we reached slightly more than 140 cm. In the Geotrench conducted at the slope we reached 135 cm (Fig 7).

The identified stratigraphic levels have been named Level 1 to Level 5, although some of them have been subdivided according to differences in sediment texture or content in organic matter. In most of the excavated area we reached the top of Level 2, while in the test pit and the Geotrench, we excavated the whole stratigraphic sequence discovered thus far, being Level 5 the basal level. The stratigraphic sequence is coherent between the different excavated areas (Fig 7).

Excavation of sediments followed standard methods in Paleolithic Archaeology. Within stratigraphic units we used small excavation tools for developing artificial layers of 3 cm thick. Artificial layers, stratigraphic units, the grid, and every archeological object or feature larger than 2 cm (lithics, bones, charcoal fragments, ocher remains and human-made structures) were georeferenced and three-dimensionally recorded using a Leica TS02 Total Station and a Leica Topographic GPS (GS16/ICG70), and orientation and dip of elongated bone and lithic products were registered. Before individually bagging archeological items, all excavation layers were digitally photographed with a Pentax K-1 II + Pentax FD24-70mm f2.8, and then recorded using a Microsoft Surface touchscreen-based PC. 3D photogrammetric models and orthomosaic plans were produced using Agisoft Metashape Professional v.1.5.2. and Adobe Creative Cloud (Photoshop v. 22.4.2. and Bridge v.11.1). The spatial distribution of items was analyzed using ArcGIS (ArcMap 10.3.1.). Stratigraphic profiles and some excavation plans were also hand-drawn. Every square meter was divided into four sectors of 0,25 sq m and sediment and items <2 cm were packed accordingly. This sediment was later floated and wet-screened at 2-, 1- and 0,5 mm mesh sieves both in the field and the laboratories of the University of Alcalá, with the main aim of collecting charcoal remains, micromammals, lithic bladelets and microdébitage.

**Fig 8:**
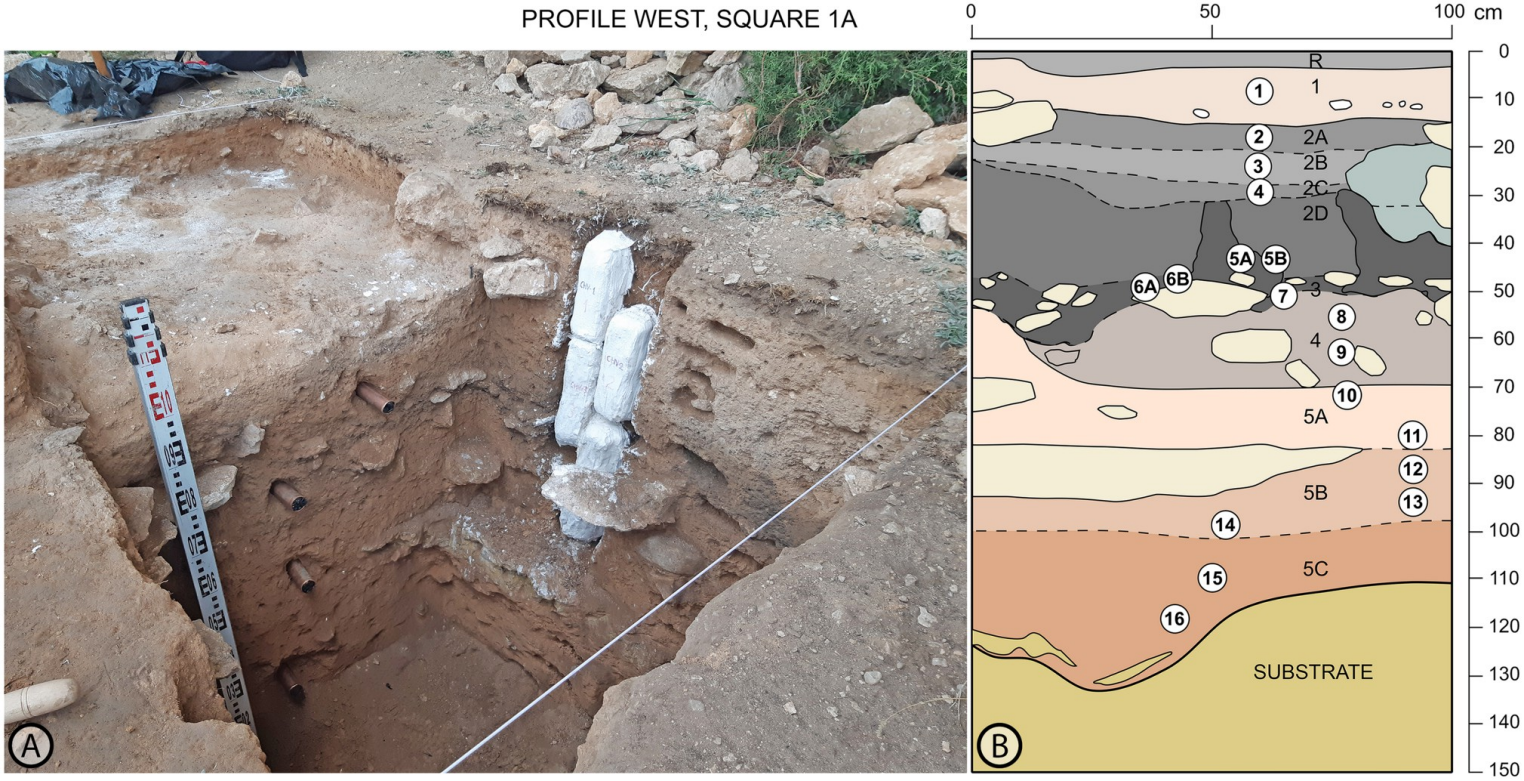
(A): Sampling for pollen analyses, micromorphology and OSL dating at profiles west and south of square 1A. (B) Stratigraphic sequence of profile west of square 1A showing the location of samples for pollen analyses.

At the end of both field seasons, stratigraphic profiles were systematically sampled for sedimentological, micromorphological, paleoenvironmental and chronometric analyses (Fig 8). Previously, some sediments and archeological features, including large charcoal pieces, were also sampled. Thus, collected samples are intended for palynological, phyolithic and anthracological analyses, as well as Optically stimulated luminescence (OSL) and radiocarbon dating, soil micromorphology and sedimentology. In this paper we present the first results on stratigraphy, sedimentology, radiocarbon dating, palynology, anthracology, zooarchaeology. and the study of archeological lithic and osseous assemblages.

### Geomorphology, stratigraphy and sedimentology

With the main aim of identifying the source of the sediments deposited at Charco Verde II, we conducted a geomorphological analysis of the landscape surrounding the site. It involved the systematic field survey of the Quaternary deposits outcropping in the area. In a second step we included all the relevant information in a GIS map, built with QGIS 3.4 software as shown in Fig 1. Stratigraphic and sedimentological analyses have been carried out in situ at the site, as well as sediment sampling of each stratigraphic level and sublevel. After the macroscopic description of the sediments in the field, the samples have been analyzed under a 10X to 40X magnification binocular microscope, with the aim of determining the lithology and texture of the sediments. We have also conducted a granulometric analysis aided by six mesh sieves ranging from 2,0 mm to 0,063 mm adding a second analysis under the microscope. Finally, we have estimated the sedimentation based on the thickness of sediment accumulated between the radiocarbon-dated samples.

### Radiocarbon dating

For radiocarbon dating, both charcoal and anthropogenically modified bone fragments collected during excavation, and hence three-dimensionally recorded and with secure stratigraphic positions, were sent to two different radiocarbon laboratories for cross-checking: the CologneAMS lab at the University of Cologne (Germany) and the Oxford Radiocarbon Accelerator Unit (ORAU) at the University of Oxford (United Kingdom). In CologneAMS, bone samples were processed by collagen extraction and charcoals were AAA (Acid–Alkali–Acid extraction) processed according to sample preparation described by Rethemeyer et al. [61]. At ORAU, extraction, purification, and dating of bone collagen were carried out following ultrafiltration methods [62], while dating of charcoal was undertaken using an ABOx-SC pretreatment [63]. For calibrating radiocarbon results we used the most recent terrestrial radiocarbon curve, IntCal20 [64]. Although dated samples do not yet cover the whole archeological sequence, we run a Bayesian model using the OxCal 4.4 online software [65], to combine the radiocarbon likelihoods with the stratigraphic position of all samples. Since each sample was three-dimensionally recorded during excavation, relationships between samples and levels (including depth within a given level) were included within the Bayesian model as prior information. We used a General t-type Outlier Model [66] with a resolution of 20 years and assigned 5% chances for each determination to be an outlier, as it is commonplace in recent research. In OxCal, commands and parameters are written in a C++ CQL (Command Query Language) [65], and Model CQL codes are provided as Supporting information (S1 Appendix). The commands used to constrain the dated events in chronological order, group them within a given stratigraphic level, and calculate a start and end boundary (Probability Distribution Functions or PDFs) to bracket each archeological episode, have been ‘sequence’, ‘phase’ and ‘boundary’ respectively [65].

### Palynology

A total of 18 palynological samples were collected from profile West of square 1A (Fig 8), where a complete sedimentary column includes five levels. Extraction followed standardized techniques for archeological sites (López-Sáez et al. 2003). The laboratory treatment was carried out in the Archaeobiology Laboratory (CSIC, Madrid) using the method by Faegry and Iversen [67] with densimetric separation of microfossils [68]. The pollen morphotypes have been established according to Faegry and Iversen [67], Moore et al. [69] and Reille [70, 71]. Pinus nigra-type pollen grains were categorized following measurements in Desprat et al. [72]. Whenever a valid sample has been given, the number of pollen grains counted or pollen sum has exceeded 200 from terrestrial plants, also housing a minimum taxonomical diversity of 20 types [73]. In the calculation of percentages, Asterioideae and Cichorioideae have been excluded from the pollen sum due to their anthropozogenic character [74]. The relative value of the excluded palynomorphs has been calculated with respect to the pollen sum. Fig 11 shows the pollen diagram elaborated with the TILIA and TGView programs [75, 76]. All samples were polleniferous. CONISS was used to assist with the biostratigraphic zonation of the pollen diagram in Local Pollen Assemblage Zones (LPAZs) according to the dissimilarity matrix of Euclidean distances and squared root transformed of percentage data [77].

### Anthracology

Charcoal remains were sampled by hand during fieldwork and by flotation in the Prehistory laboratory of the University of Alcalá. 59 samples from levels 1 to 5, basically single fragments of charcoal hand-collected in situ during excavations, have been studied in the Environmental Archeology laboratory of the CSIC (Madrid). A total of 383 fragments of carbonized wood were localized, of which 331 were identified to taxon and 52 remained unidentifiable. Identification of taxa was carried out following standard procedures. The anatomical patterns of each wood species were observed along three sections (transversal, longitudinal tangential, and longitudinal radial) using a reflected light microscope equipped with light field/dark field and objectives of 50 ×, 100 ×, 200 × and 500 ×. Archeological samples have been compared with modern woods as well as with wood anatomy atlases [78–80].

### Zooarchaeology

2,509 faunal remains from levels 1 to 5 of Charco Verde II, coming from both the 2020 and 2021 seasons, were subject to zooarcheological and taphonomic analyses. 1,709 of them come from Level 1, which is the only layer which has been completely excavated in all squares opened to date. Studied remains included both identifiable and unidentifiable fragments and their taxonomic identification were based on reference material held at the Prehistory Department of the Complutense University of Madrid (Spain), as well as on reference atlases [81–83]. When the identification was not feasible, epiphyses, axial and shaft fragments were assigned to three animal weight/size classes: 1) small-sized carcasses, < 100 kg (e.g. Capra pyrenaica, Rupicapra rupicapra), 2) medium-sized carcasses, > 100–350 kg (e.g. Cervus elaphus) and 3) large-sized carcasses, > 350 kg (e.g. Equus ferus, Bos primigenius).

The estimation of NR (Number of Remains) and MNI (Minimum Number of Individuals) were used to quantify the faunal remains and determine the most appropriate features of the faunal taxonomic distribution. NR included determinable and indeterminable bone remains and MNI is based on Brain’s method [84], which uses bone laterality and estimated age. Furthermore, skeletal profiles and MNI consider shaft thickness, section shape and medullar surface properties (see [37] for detailed methodological descriptions).

Mortality patterns were divided into (1) infants (individuals dead before the first six months of life, as shown by the absence of the first permanent molar), (2) juvenile-prime adults (individuals showing the second permanent molar and deciduous p4) and (3) adults (those with all permanent teeth). Age profiles were estimated from tooth crown wear and the emergence of the teeth according to Stelle [85] for deer, Pérez Ripoll [86] for ibex and Levine [87] for Equus.

A systematic observation of bone surfaces to explore the presence of cut, percussion and tooth marks, as well as thermal alterations, was also carried out with 10X-20X hand lenses and different lighting, as well as with a Leika 10-60X binocular loupe. Our diagnostic criteria for cut, percussion, carnivore and rodent marks are those defined by Potts and Shipman [88], Blumenschine & Selvaggio [89] and Fernández-Jalvo & Andrews [90]. Weathering stages were also observed following Behrensmeyer [91] to estimate the bone subaerial time exposure. Water effects on bone surfaces were estimated according to the presence of abrasion, polishing, rounded bones, and carbonates following Parson and Brett [92], Cáceres [93] and Yravedra [94]. Biochemical alterations, including both pre- and post-depostional biological processes produced by insects, fungi, bacteria or plants, were estimated according to Fernández-Jalvo & Andrews [90]. To differentiate between green and dry fractures on long bones we analyzed shafts larger than 30 mm following Villa & Mahieu’s [95] criteria.

### Study of lithic and osseous assemblages

Lithic and osseous assemblages recorded throughout the sedimentary sequence of Charco Verde II were hand-collected during excavation fieldwork and, in the case of ˃2 cm artifacts, after wet-screening of sediments at 2-, 1-mm and 0.5-mm mesh sieves. After manual cleaning and removal of adhering concretion, archeological assemblages, including lithic artifacts, osseous tools and personal ornaments made on shells, were studied at the Prehistory Laboratory of the University of Alcalá. Concerning lithics, for the basic techno-typological characterization we followed the *chaîne opératoire* or ‘operational sequence’ approach [e.g. 96, 97], combined with the ‘Technological organization’ approach aimed at examining links between technology, environmental constraints, and settlement and mobility patterns [e.g. 98–100]. We assigned each lithic artifact to one of the three *chaîne opératoire* stages commonly recognized in the literature (S2 Appendix). Thus, cortical flakes, preparation products and tested cores were assigned to the initialization stage or phase I; raw blanks, core maintenance by-products and productive cores to the exploitation stage or phase II; and retouched blanks, burin spalls, and exhausted cores to the consumption and abandonment stage or phase III. Bladelets are ≤ 2 cm long. Knapping waste, composed of chunks and microdébitage, was included in the exploitation stage. Microdébitage includes flake fragments and chips (i.e. trimming flakes) whose maximum length is smaller than 1 cm, as well as shatter, considered as small (≤ 1 cm) angular debris. Chunks are fragmented angular > 1cm pieces that do not clearly show ventral or dorsal surfaces. Classification of retouched products was based on the classic type-list proposed by Sonneville-Bordes and Perrot for the Upper Paleolithic [101], modified when needed. For further chrono-cultural contextualization we considered recent literature on Magdalenian lithic technology and typology in southwest Europe [e.g. 102–107].

Antler tools were described according to current classifications in the Upper Paleolithic repertoires of southwest Europe [see 108 for references].

## Results

### Stratigraphy and sedimentology

**Fig 9:**
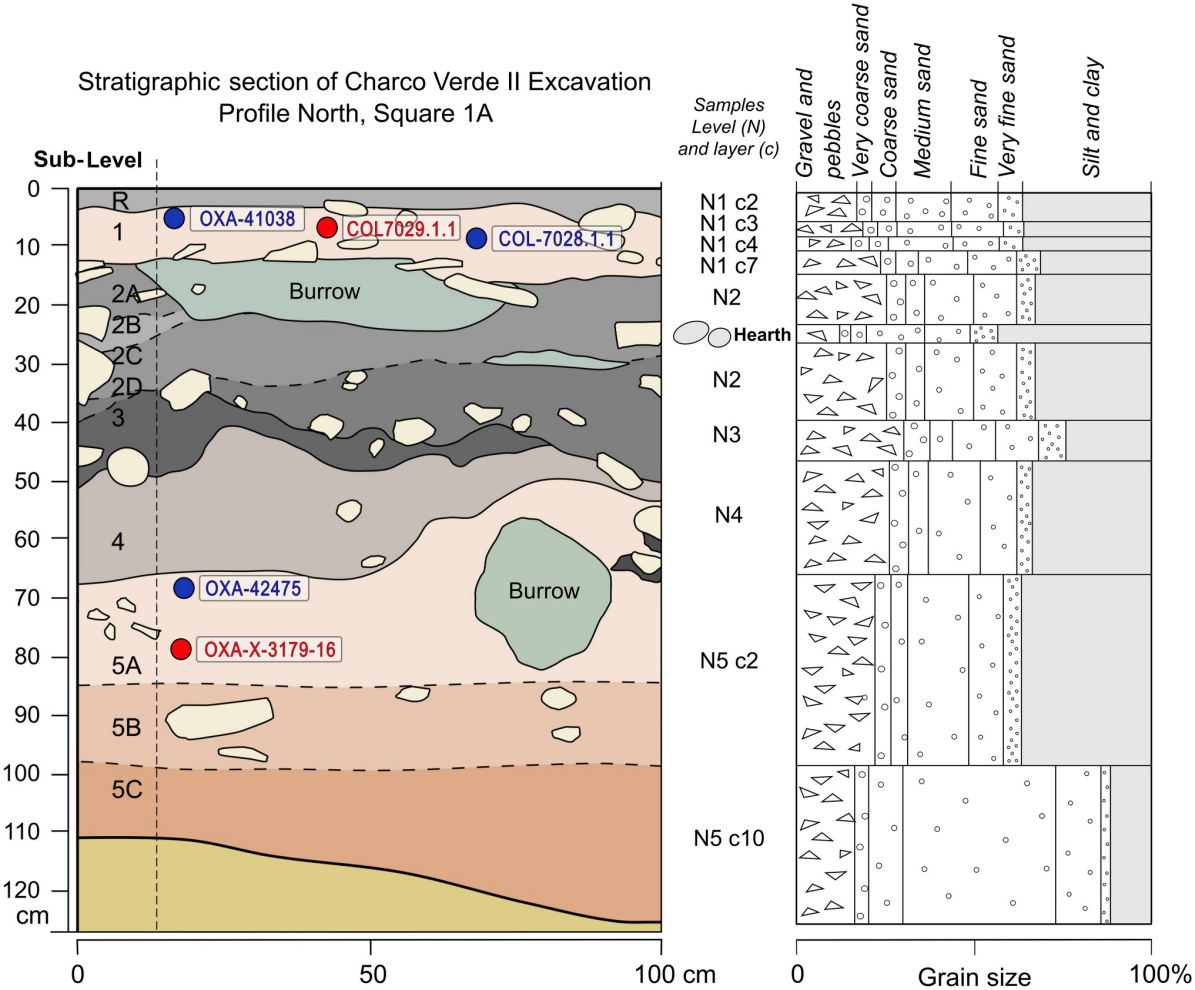
Detailed stratigraphy and grain size analysis of the sediments sampled in square 1A of Charco Verde II. It is shown the schematic location of samples used for radiocarbon dating (charcoal in blue, bone in red).

The archeological sediments consist of poorly sorted silty sand, with carbonate grains, and abundant large planar and angular to sub-rounded dolostone blocks and cobbles. The angular blocks mainly come from the erosion and weathering of the dolostone wall, and the sub-rounded ones derive from the upper hill slope (Fig 9). The deposit is fairly massive, but clasts lie horizontally, and diffuse lamination is preserved in some of the sublayers, thus suggesting an internal structure. Together with carbonate components, the sediments contain scattered charcoal fragments, rounded quartzite clasts and bone fragments. In some cases, these bone remains show deeply altered cortical surfaces due to root marks and dissolution. Many of the observed fractures are longitudinal and oblique. There is an increase in the amount of quartz and quartzite rounded grains towards the base of the deposit, reaching more than 90% of the components in the finer grain sizes. Thin calcite cemented rhizolith tubes are common in the upper part of the sequence.

We have distinguished five different stratigraphic levels based on grain size variation and the relative content of organic matter (charcoal fragments). These levels were named 1 to 5 from top to bottom (Fig 9). Levels 1, 2 and 3, and to a lesser extent level 4, contain larger amounts of ash, charcoal remains and burnt stones. As recorded in the excavated areas conducted to date (Fig 6), at least levels 1 and 2 are spread all around the near sedimentary deposit, from the rock wall to the talus, where they dip outward.

Apart from the archeological levels 1 to 5, we also identified a superficial level, 6 to 18 cm thick, deeply reworked, with a large amount of organic matter and sub-rounded clasts, that we named Level R. Despite its sedimentological features being very similar to Level 1, it contains a mixture of both ancient lithics and modern remains. The sedimentological characteristics of the in situ archeological levels are the following:

Level 1: 10–16 cm thick in the flat area of the deposit and up to 42 cm in the Geotrench near the slope. Light beige carbonate silty sand, mainly medium to fine grain size, with a large amount of angular dolostone blocks. There are a small number of micromammal bones, charcoal fragments (smaller than 1 cm) and rhizoliths (as fine tubes of well cemented sand). There are white flint lithic debris. The lower contact with level N2 is sharp, but the upper one is diffuse mixed with reworked level R.

Level 2: 8–42 cm of a thick, irregular silty sand bed with a light grey to almost black color showing some sub-levels depending on the amount of charcoal micro fragments (usually above 0,5 mm in size). There are anthropogenic concentrations of quartzite rounded cobbles and dolomite blocks larger than 15 cm with a higher amount of charcoal fragments. Rhizoliths and bone fragments are very common, as well as a high proportion of the coarser sediment grains. The lower contact is sharp and erosive with the underlying bed of level 3.

Level 3: 0–11 cm of grey, medium to fine sand with a larger amount of dolostone blocks and gravels. It also includes root marks and bone fragments. Despite its grey color, the amount of charcoal seems to be lower than in level 2 under the lens -probably being finer size ashes-, but isolated big centimetric charcoal fragments have also been found in it. The number of lithic debris is higher, with white and beige flint being well represented. Finer grain size sand shows a light increase in quartz and quartzite grains. Lower contact with level 4 is sharp and erosive.

Level 4: 3–16 cm of beige-grey silty sand, very similar to level 3, but showing an increase in dolostone rounded clasts. Rhizoliths are highly abundant, especially in diameters larger than 0.5 mm. White and beige flint lithic debris are common, as well as bone fragments. The amount of charcoal is low, but quartz and quartzite, even though they are minorities, increase at the bottom of this level.

Level 5: 45–65 cm of a thick, moderately sorted sand level, beige in color, in a finning upward sequence, with abundant large dolostone blocks. Textural changes allowed us to subdivide it in four sublevels. The proportion of silt increases towards the top, but clay is present in the lower part of the level too. The upper part shows carbonate nodules, abundant rhizoliths, but much lower amounts of bone fragments and charcoal than in the previous levels. Angular gravel and small block-size dolostone clasts are proportionally reduced while most rounded clasts are abundant, suggesting more slope or even alluvial influences. Quartz and quartzite are also more abundant, reaching 10% in the coarser grain size and 70–90% in the finer ones. The lower part of Level 5, as recorded in the Geotrench, fills a dolostone paleorelief and is composed by better sorted, loose, medium to coarse quartz sand. Lithic fragments and charcoal remains are very scarce.

The whole sequence shows bioturbations filled with sediments, consisting of large burrows probably caused by micromammals. The stratigraphic integrity of the deposit is deeply altered at these concrete points, but the main part of it remains unaltered. The upper part of the deposit also shows some mud insect nests. These post-depositional features are relatively easy to recognize and allow us to interpret the intact remains of the archeological deposit as being in a primary position. The micromorphological analyses to be conducted soon are expected to provide further evidence in this regard.

The sedimentological analysis and the geometry of the layers, dipping parallel to the slope of the site, confirm that it was formed as a hillslope deposit at the foot of the dolomitic rock wall. The sediments come from both the wall itself due to frost weathering and the slope above it, mixing angular clasts with more rounded dolomite clasts and small size carbonate and quartz sand grains. The height above the current river and the lack of quartzite clasts, found into the lower Piedra River terrace, discard the fluvial contribution to the site formation, at least above the lowermost quarzitic sandy beds of Level 5. Considering the radiocarbon dates available, the sedimentation rate shows a clear decrease upward, from 0.73 mm/yr in Level 5 to 0.02–0.03 mm/yr in Level 1, and 0.131 mm/yr in between. This could be due to a decrease in the physical and chemical weathering of the bedrock due to a progressive loss of effective moisture in the environment along the time of deposition [109, 110], but the lack of Holocene deposits on top of the archeological levels suggests other factors leading to landscape stability. These factors can include changes in the shape of the rock shelter and upper slope, increasing climate stability, greater vegetation cover fixing the soil and soil thickening [e.g. 111–113]. Therefore, human groups occupied this low sedimentation rate platform on top of the hillslope colluvium.

### Radiocarbon dating

**Fig 10:**
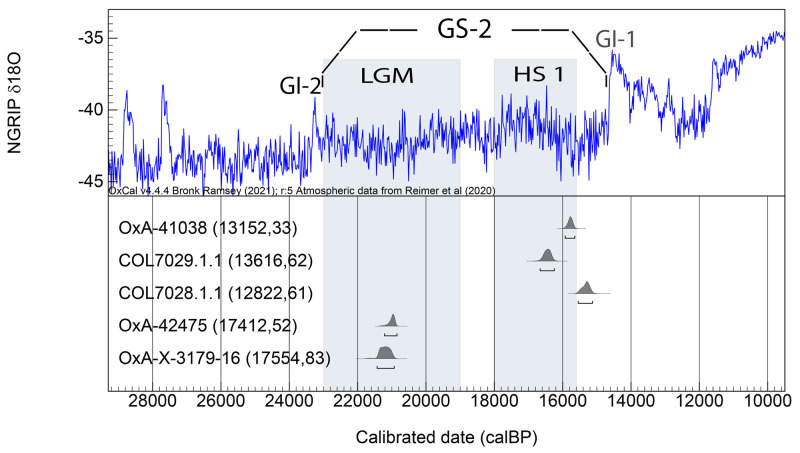
Modelled radiocarbon dates for levels 1 and 5 of Charco Verde II (see Table 1). Results are plotted against the δ18O record of the NGRIP ice core, indicating Greenland Interstadials 2 and 1 (GI 2 & GI 1), Greenland Stadial 2 (GS 2) [10], and the chronology of the LGM (sensu [114]) and HS1 [12]. ^14^C dates are shown in parentheses.

As a first step for delimiting the chronological setting of the archeological sequence preserved at Charco Verde II, we attempted the radiocarbon dating of five samples: two charcoal pieces and one bone coming from the top of the sequence (Level 1), and one charcoal piece and one bone fragment from the base (Level 5). Both bones presented anthropogenic fractures. Obtained results (Table 1) place the youngest occupation phase of the site between 15.1 and 16.6 ka cal BP (Level 1) and the oldest between 20.8 and 21.4 ka cal BP. As the dated samples show high agreement indexes, these time frames remain the same after modeling (Fig 10 and S1 Appendix) There are, however, some uncertainties posed by the three dates obtained for Level 1, as there is no overlapping among them, thus suggesting that this level could be a palimpsest reflecting several periods of human occupations spanning as much as ∼1500 years. Whether this is the case, or the issue is best explained by contamination of samples, is a question that requires further research, including more excavation, dating, and modeling when a larger sample dataset is available. Concerning Level 5, although OxA-X-3179-16 has been considered potentially inaccurate by ORAU, due to a permil difference between the delta 13C measured on the AMS and IRMS which was slightly above (0.8) the limit accepted by the lab, in chronostratigraphic and archeological grounds the date is fully consistent.

**Table 1:** Radiocarbon determinations obtained for the top (Level 1) and the base (Level 5) of the archeological sequence recorded at Charco Verde II. C^14^ dates were calibrated with OxCal 4.4 [65] using IntCal20 [64].

Level	Sample ID	Material	Lab ID	C^14^ BP	δ13C (‰)	cal BP (95,4%)
1	CHAR20-1	Charcoal	OxA-41038	13152 ± 33	-23.6	15925–15645
1	CHAR20-9	Bone	COL7029.1.1	13616 ± 62	-18,0	16660–16245
1	CHAR20-21	Charcoal	COL7028.1.1	12822 ± 61	-22,5	15547–15125
5	CHAR21-5.1–20	Charcoal	OxA-42475	17412 ± 52	-22.1	21212–20848
5	CHAR21-5.2-BN	Bone	OxA-X-3179-16	17554 ± 83	-19.9	21430–20930

Therefore, despite these minor uncertainties, which for now hamper building a very precise chronometric sequence for the human occupations registered at Charco Verde II, it is sufficiently clear that the rock shelter was occupied by humans starting at least during the early phases of the Magdalenian (Lower or Archaic Magdalenian as recorded in Level 5), while the last recorded occupations were produced during advanced phases of this technocomplex (Middle or Upper Magdalenian as recorded in Level 1). As we will discuss below, techno-typological features of the lithic and bone industries gathered at Level 1 match these chrono-cultural assignments.

It is also relevant that both phases started and developed during Greenland Stadial 2 (GS-2) and were not coincident with any interstadial phase as recorded in the Greenland ice-core records [10] (Fig 10). More significantly, the occupation recorded at Level 5 occurred at the height of the LGM *sensu stricto* (i.e., as classically defined around 21 ka cal BP [114]), thus suggesting that harsh climatic and environmental conditions were dominant during this timeframe. Furthermore, dates obtained for Level 1 also link at least part of its occupations to cold climates and dry environments, as OxA-41038 and COL7029.1.1 match the last phases of HS 1 [12], while COL7028.1.1 is slightly younger, but still falls within GS-2 (Fig 10). Below we will discuss how these paleoclimatic inferences based on chronometric results and off-site archives are complemented by on-site paleoecological data collected directly at the Charco Verde II sequence.

### Pollen

**Fig 11:**
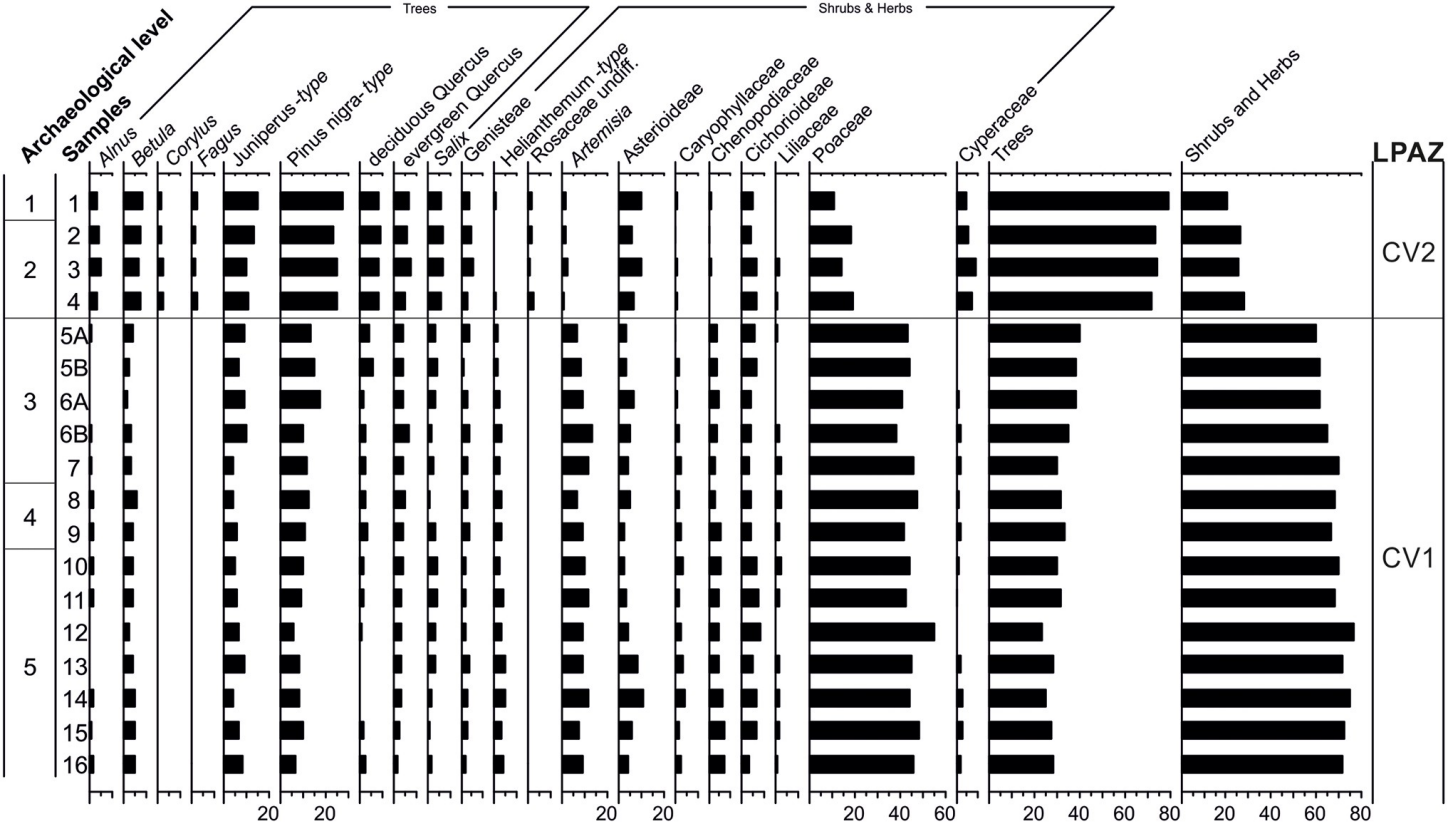
Percentage pollen diagram from the Charco Verde II sequence.

For the Charco Verde II sequence, 18 pollen spectra were analyzed and 20 taxa were identified. To facilitate description and interpretation of the pollen diagram with respect to vegetational changes, two Local Pollen Assemblage Zones (LPAZs) were established (Fig 11). These zones denote significant changes in the pollen composition and represent major changes in vegetation.

LPAZ CV1 (samples 5A to 16, corresponding to archeological levels 3, 4 and 5) reflects an open landscape dominated by steppic grasslands (dominance of Poaceae and steppic plants such as *Artemisia*, Chenopodiaceae and *Helianthemum*) in the surroundings of the site, with sparse leguminous scrubland (Genisteae). Trees (*Pinus nigra*, *Alnus*, *Betula*, deciduous *Quercus*, *Salix*) would have been scattered over the territory, or would have formed small woods dispersed over the steppic landscape. These data are in agreement with arid and very cold conditions corresponding to the end of the LGM (GS-2b stadial) at ~ 21–17 ka cal BP [8], as also shown by low values of hygrophytic herbs (Cyperaceae). The preponderance of junipers (*Juniperus*) would also characterize this cold, continental phase [115], although pollen data show the relative importance of mountain pine forests at this time [116].

LPAZ CV2 (samples 1 to 4, corresponding to archeological levels 1 and 2) becomes dominated by more dense forest vegetation. The arboreal pollen sum is mainly made up of abundant pollen-producing cold-adapted tree taxa, such as *Pinus nigra*, *Betula*, *Alnus* and *Salix*, indicating the presence of open pine forests and sparse riparian woodlands in the surroundings of the site [117]. However, mesophilous elements such as deciduous and evergreen oaks, and even others such as beech (*Fagus*) or hazel (*Corylus*), which appear for the first time in the sequence, are also found. Riparian trees (*Alnus*, *Salix*) also increase their percentages in this pollen zone, as do wet grasses of Cyperaceae, while steppic elements (*Artemisia*, Chenopodiaceae, *Helianthemum*) and Poaceae are significantly reduced. *Juniperus* and Genisteae are continuously present with increasing values. Both pollen taxa produce large quantities of pollen, but low amounts of pollen are usually found in fossil records, suggesting the development of broom communities and junipers (probably *Juniperus thurifera*) within the open pine woodlands [118]. This pollen zone, roughly dated at ~ 17–15 ka cal BP, could correlate with the GS-2.1a stadial, also known as Oldest Dryas, which includes Heinrich Event 1. This phase, also known as the “Mystery Interval” [119], shows great complexity within the paleoclimatic reconstructions of the Iberian Peninsula: in some cases it is shown as a colder and drier period even than the LGM [23]; in other ones it shows more temperate conditions allowing for glacial refugia for vegetation [8, 118]. The latter would be the case for the site under study, suggesting that the shading effect of the last deglaciation was less important in this area, which allowed for tree development [120] despite its harsh biogeographic setting.

### Wood charcoal

Fragments of wood charcoal have been found in all layers throughout the sequence of Charco Verde II. However, levels 1 and 2 have shown higher quantities of wood remains, as they have been excavated in a larger area, while levels 3 to 5 are only known in the 1x1 m test pit conducted at square 1A (Figs 6 & 8). Among the identified wood fragments, taxonomic diversity is low (Table 2), being most of the fragments (∼90%) assigned to *Juniperus* sp. In Level 2 a fragment of cf Rosaceae is also found, while Level 1 has shown pine wood, deciduous *Quercus*, Rosaceae and a possible *Salix/Populus*. Together with these identified taxa, there are unidentified fragments, or fragments just identified as conifers. This is due to the high degree of vitrification of charcoals, which in some cases hampers a proper identification of the diagnostic anatomic features.

**Table 2:** Wood charcoal identified in Charco Verde II.

Level	*Juniperus* sp	*Pinus* sp	QsQ	Rosaceae tp *Prunus*	cf Rosaceae	cf *Salix/Populus*	Gimnosperm	Unident.
1	170	3	3	3	1	6	3	24
2	111				1			12
3	5						1	
4								2
5	10						14	14

When considered together with the pollen results, the wood charcoal data suggest that hunter-gatherers settled at Charco Verde II selected mostly *Juniperus* wood for producing fire, as trees of this type were accessible in the surroundings of the site throughout the sequence. In the cold and arid phase documented for LPAZ CV1 (archeological levels 3 to 5), *Juniperus* was probably an accessible wood in an otherwise open and steppic landscape. As climate and environmental conditions ameliorated in LPAZ CV2, other woods were added to the repertoire, but *Juniperus* remained the most used. This persistence is best explained due to the high hardiness and resistance of this wood, which is optimal for fire-making [121, 122]. Some of the species of the *Juniperus* genre, such as savin juniper, are very well adapted to cold temperatures, and thus their presence in the sequence of Charco Verde, developed throughout the GS-2 and including part of the LGM and HS 1, is to be expected.

### Macrovertebrates

Most of the 2,509 analyzed bone fragments throughout the Charco Verde II sequence present a high degree of fragmentation, and hence only 3.71% of them were identified to taxon. However, although 96.29% of remains are thus indeterminate (being above 85% in all levels), 7.69% among them could be assigned to either small, medium or large-sized animals. To certain degree, this high number of indeterminate remains is due to the small size of bone fragments. For Level 1, 85.3% of the remains are smaller than 3 cm, and this percentage is even higher in other levels (Table 3).

**Table 3:** Taxonomic representation of macrovertebrates remains (NR) at the Charco Verde II sequence.

Taxon/Level	1	%	2	%	3	%	4	%	5	%	Total
*Equus*	63	3.7	4	1.3		0.0	1	0.4	1	0.5	69
*Bos/Bison*		0.0	1	0.3		0.0		0.0		0.0	1
*Capra pyrenaica*	5	0.3	1	0.3		0.0	2	0.8	5	2.5	14
*Cervus elaphus*		0.0	2	0.6		0.0		0.0	1	0.5	3
*Carnivora indet*	5	0.3		0.0		0.0		0.0		0.0	5
Salmonid		0.0		0.0		0.0	1	0.4		0.0	1
Indet	1485	86.9	281	89.8	53	100.0	225	95.0	186	94.4	2230
Indet. small size	62	3.6	18	5.8		0.0	8	3.4	4	2.0	92
Indet. medium size	7	0.4	2	0.6		0.0		0.0		0.0	9
Indet. Large size	81	4.7	4	1.3		0.0		0.0		0.0	85
**Total NR**	1709	100.0	313	100.0	53	100.0	237	100.0	197	100.0	2509
Total NR <3cm	1458	85.3	288	92.0	53	100.0	212	89.5	193	98.0	2204
Total NR 3.1–5 cm	110	6.4	15	4.8	0	0.0	3	1.26	3	1.52	131
Total NR >5.1 cm	141	8.3	10	3.2	0	0.0	22	9.3	1	0.5	174

Among the identified species, the most common are animals adapted to open landscapes and grassland areas. Thus, ibex, and especially horses, are clearly dominant throughout the sequence. Other less frequent species include red deer in levels 2 and 5, large bovid (either bull or bison) in Level 2, salmonid in Level 4, and indetermined carnivores in Level 1 (Tables 3 & 4). Mortality patterns are dominated by adult individuals, except for Level 4, where juveniles are more frequent (Table 4).

**Table 4:** Taxonomic representation of macrovertebrates remains at the Charco Verde II sequence according to MNI (Adult/Juvenile/Infant when specified).

MNI/Level	1	%	2	%	3	%	4	%	5	%	Total
*Equus*	2	50.0	1	20.0		0.0	0/1/0	33	1	33	5
*Bos/Bison*		0.0	1	20.0		0.0		0.0		0.0	1
*Capra pyrenaica*	1	25.0	1	20.0		0.0	1/1/0	67	1	33	3
*Cervus elaphus*		0.0	1	20.0		0.0		0.0	1	33	2
*Carnivora indet*	1	25.0	1	20.0		0.0		0.0		0.0	2
**Total**	4	100.0	5	100.0	0	100.0	3	100.0	3	100.0	13

Concerning bone surface preservation, besides the mentioned intense breakage degree, the main modification affecting the assemblage are biochemical alterations, especially vermiculations, which are found in the 100% of the remains. This is an important factor hampering the identification of butchery marks, as only four bones in Level 1 have shown cut marks (3 horse remains and one small-sized animal). Other modifications suggesting anthropogenic activity, identified at the macroscopic level, include thermal alterations in 20.9% of the bone remains from Level 1 (Table 5).

**Table 5:** Bone surface modifications recorded at the Charco Verde II macrovertebrates assemblages according to the number of remains.

Surface modifications/Level	1	%	2	%	3	%	4	%	5	%	Total
Biochemical	1709	100.0	313	100.0	53	100.0	237	100.0	197	100	2509
Weathering	1	0.1		0.0		0.0		0.0		0.0	1
Hydrological alteration	2	0.1	1	0.04		0.0		0.0		0.0	3
Rodent tooth mark	1	0.1		0.0		0.0		0.0		0.0	1
Cut marks	4	0.2		0.0		0.0		0.0		0.0	4
Thermal alteration	357	20.9	8	2.6	1	1.9	2	0.0	1	0.52	369
Green fractures in long bone shafts	137	66.8	26	96.3		0.0	26	100.0	3	100.0	192
Dry fractures in long bone shafts	68	33.2	1	3.7	4	100.0		0.0		0.0	73
**Number of remains (NR)**	1709	100.0	313	100.0	53	100.0	237	100.0	197	100.0	2509
NR <3cm	1458	85.3	288	92.0	53	100.0	212	89.5	193	98.0	2204
NR 3,1–5 cm	110	6.4	15	4.8	0	0.0	3	1.26	3	1.52	131
NR >5,1 cm	141	8.3	10	3.2	0	0.0	22	9.3	1	0.5	174

Despite the limitations posed by the poor preservation of the bone surfaces and the intense breakage degree of the assemblages, some traits enable us to suggest that the Charco Verde II faunal accumulation is of anthropogenic nature throughout the sequence, and especially in Level 1, which is the only one with a significative number of remains to date. In this level, the high quantity of thermal alterations and green fractures in long bone shafts, together with the presence of a limited number of cut marks (Table 5) points to the anthropogenic nature of the accumulation. Furthermore, the absence of carnivore marks in the bone assemblage also points to humans as the only agent involved, being the activity of other animals absent or insignificant. In this regard, the scarcity of weathering modifications and hydrological alterations, such as abrasion, polishing or rounded bones, (Table 5), suggest that the bone assemblages were not exposed for a long time. Only the presence of rodent tooth marks in one remain coming from Level 1 suggest that other agents could interact with the bone carcasses once the human groups left the site.

### Lithic assemblages, osseous tools and personal ornaments

All stratigraphic levels recorded at Charco Verde II have shown archeological remains, from level R to Level 5. The scarce extension excavated to date, limited to 5.5 sq meters for Level 1 and the very top of Level 2, and just 1 sq meter for the rest of the sequence (as conducted at the test pit of square 1A) (Figs 6 & 8), entails that the number of archeological remains is still low. However, artifacts recorded at Level 1, composed of knapped lithic products, faunal remains (bone, antler and shells), osseous tools and wood charcoal pieces, suffice to propose a solid chrono-cultural assignment for the human occupation associated to it. Below we present the first results obtained in the study of these assemblages.

**Fig 12:**
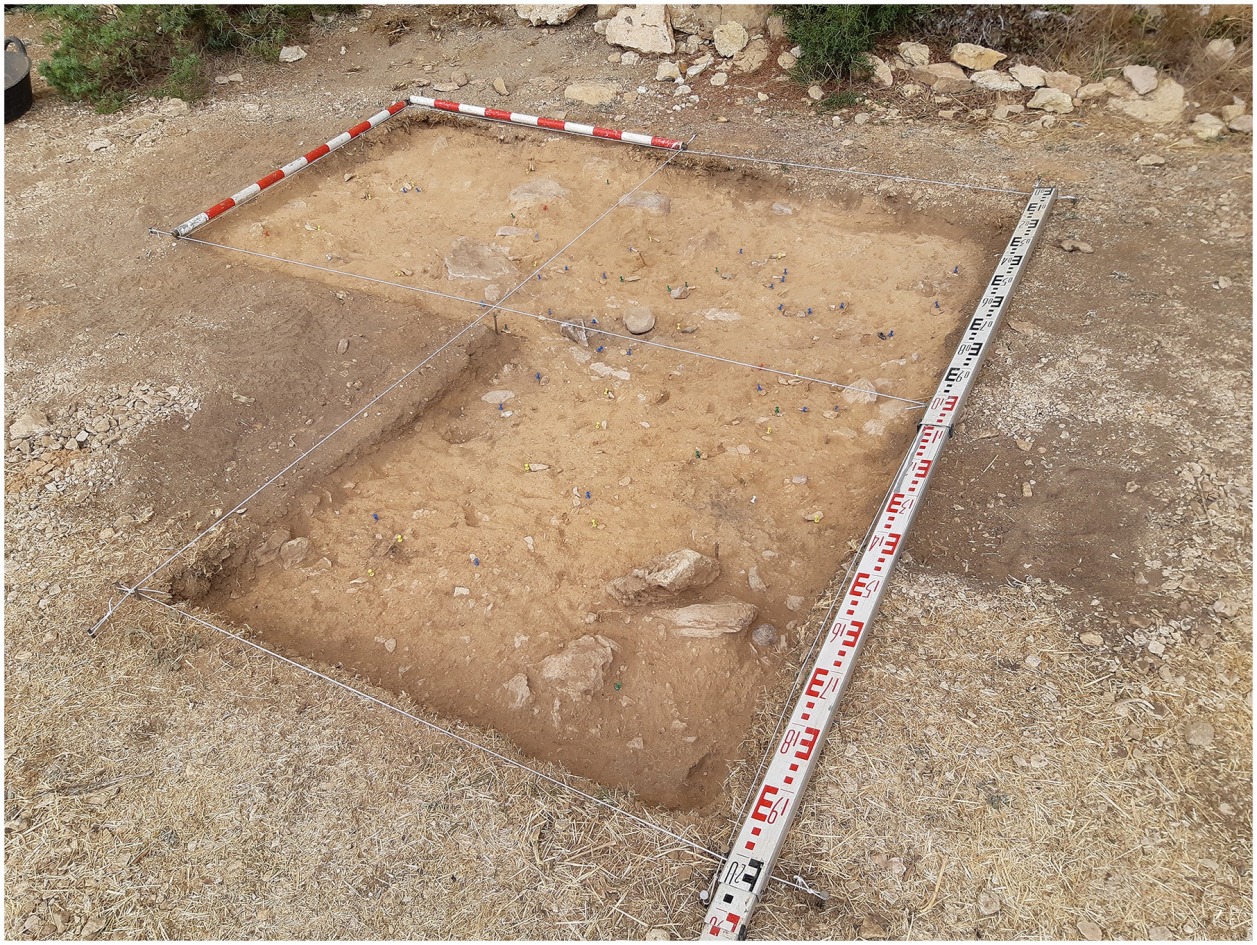
Squares 1A, 1B and 2A excavated at a central location of the Charco Verde II deposit, after excavation of layer 2 of Level 1.

**Fig 13:**
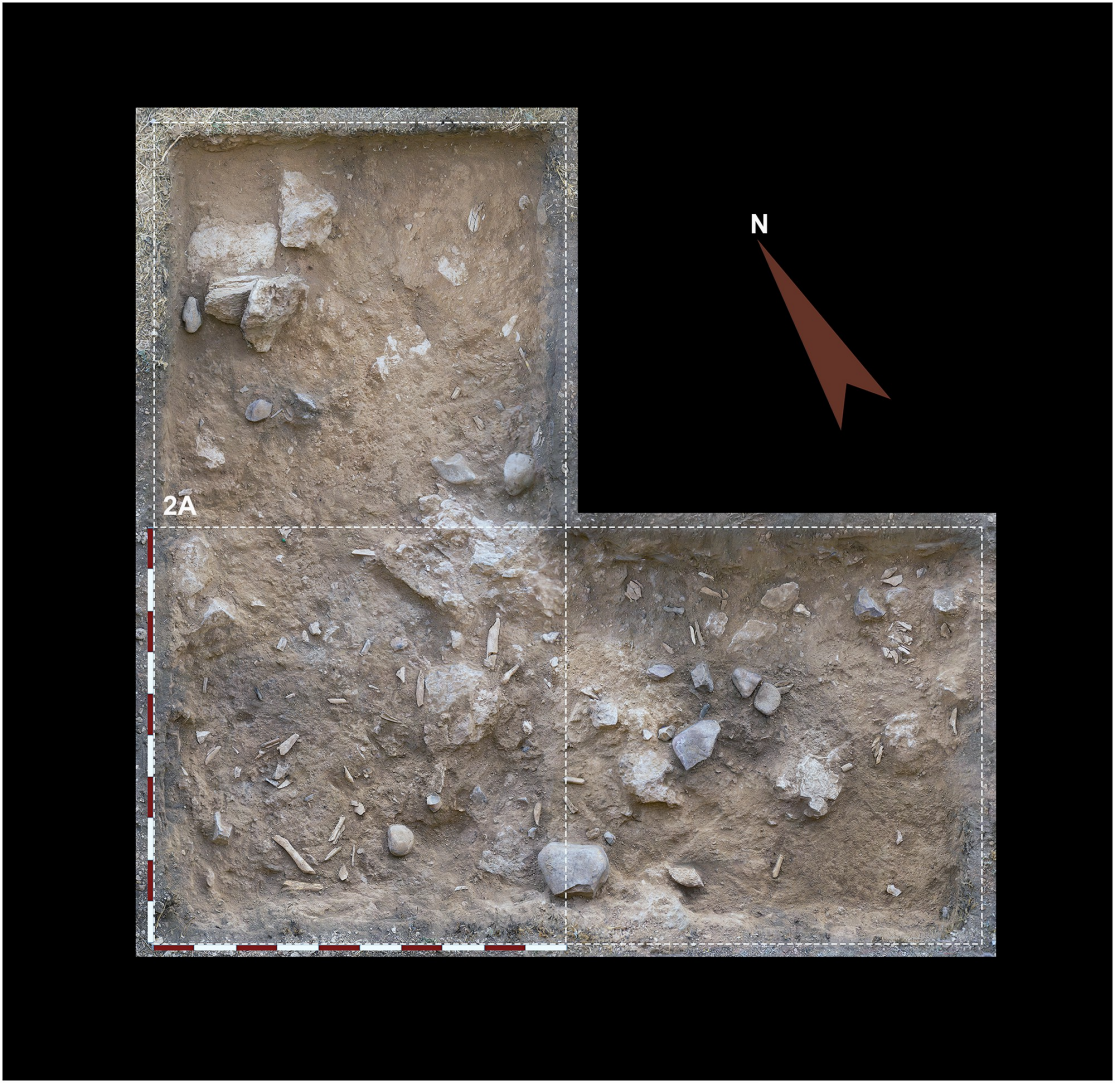
Orthomosaic showing squares 1A, 1B & 2A after excavation of layer 4 of Level 1.

The first aspect to consider is the relatively optimal degree of conservation of the archeological record, given that Level 1 is found less than 20 cm below the current ground surface (Figs 8 & 12). This includes the spatial distribution of archeological remains, which, although not yet confirmed by microspatial or taphonomic analyses, seems not to have suffered significant post-depositional disturbance (Figs 12 & 13). This is suggested by the high density of archeological materials, the absence of preferential orientation patterns of elongated remains, the presence of probable fire structures and the recording of at least two refitting sequences clustered at two different locations of the excavated areas (two and three refitted pieces respectively). Preservation of the lithic product’s surfaces is also good, although a relevant number of flint specimens exhibit a white patina or certain degree of dehydration (15.16% of the total assemblage) due to chemical processes (Fig 14.5 & 14.7). By contrast, most of the bone and antler remains are heavily affected by surface alterations, including vermiculations, and biochemical processes (Fig 15), as previously described for the complete faunal assemblage.

**Fig 14:**
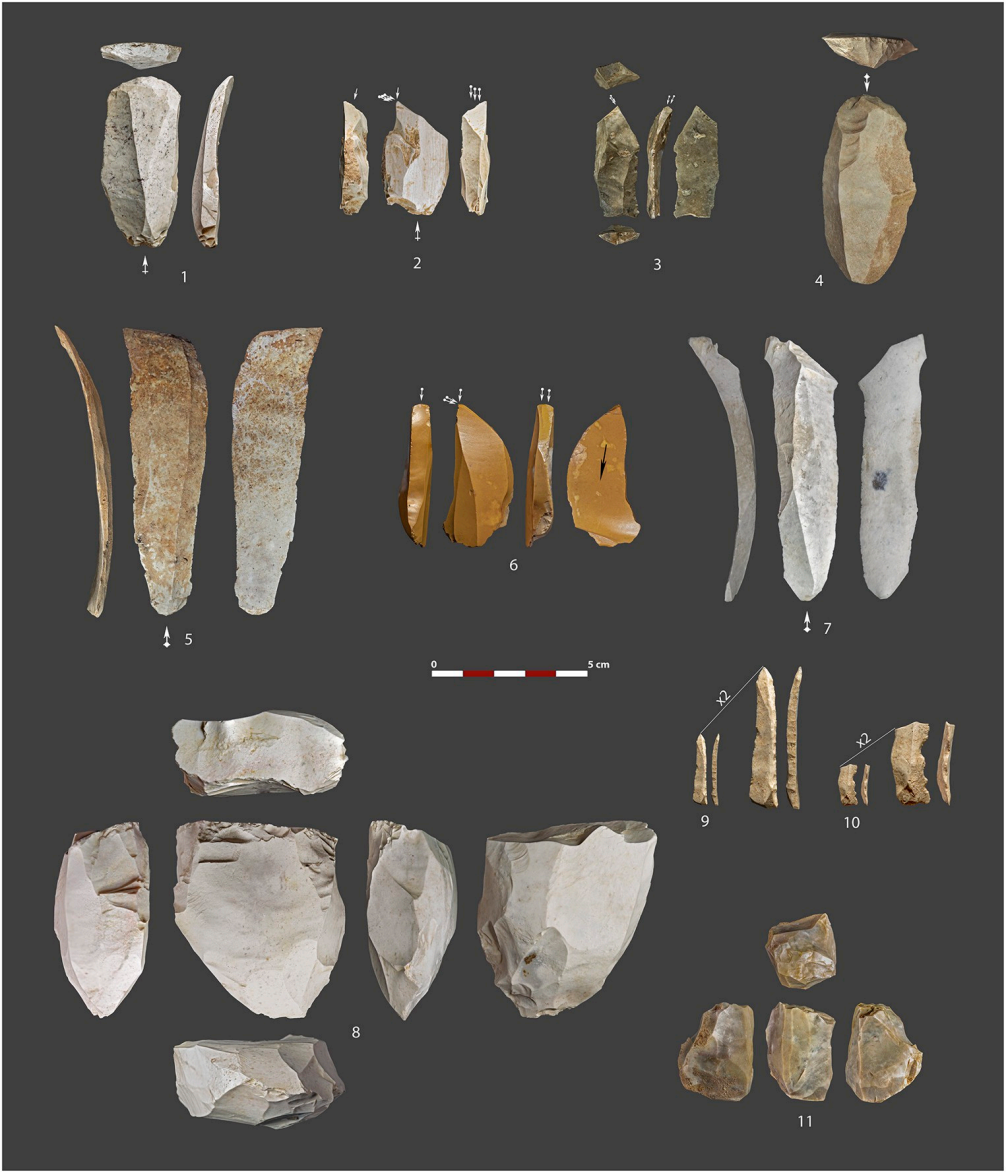
Selection of lithic artifacts collected at the Charco Verde II site. All come from Level 1 except number 3, which was found on the ground Surface of the archeological deposit, and number 6, recorded at the fluvial terrace below the slope. 1 & 4: Endscrapers on blades. 2, 3 & 6: Canted dihedral burins. 5 & 7: Large blades. 8: Unidirectional blade core. 9: Backed bladelet. 10: Denticulated backed bladelet. 11: Unidirectional bladelet core.

**Fig 15:**
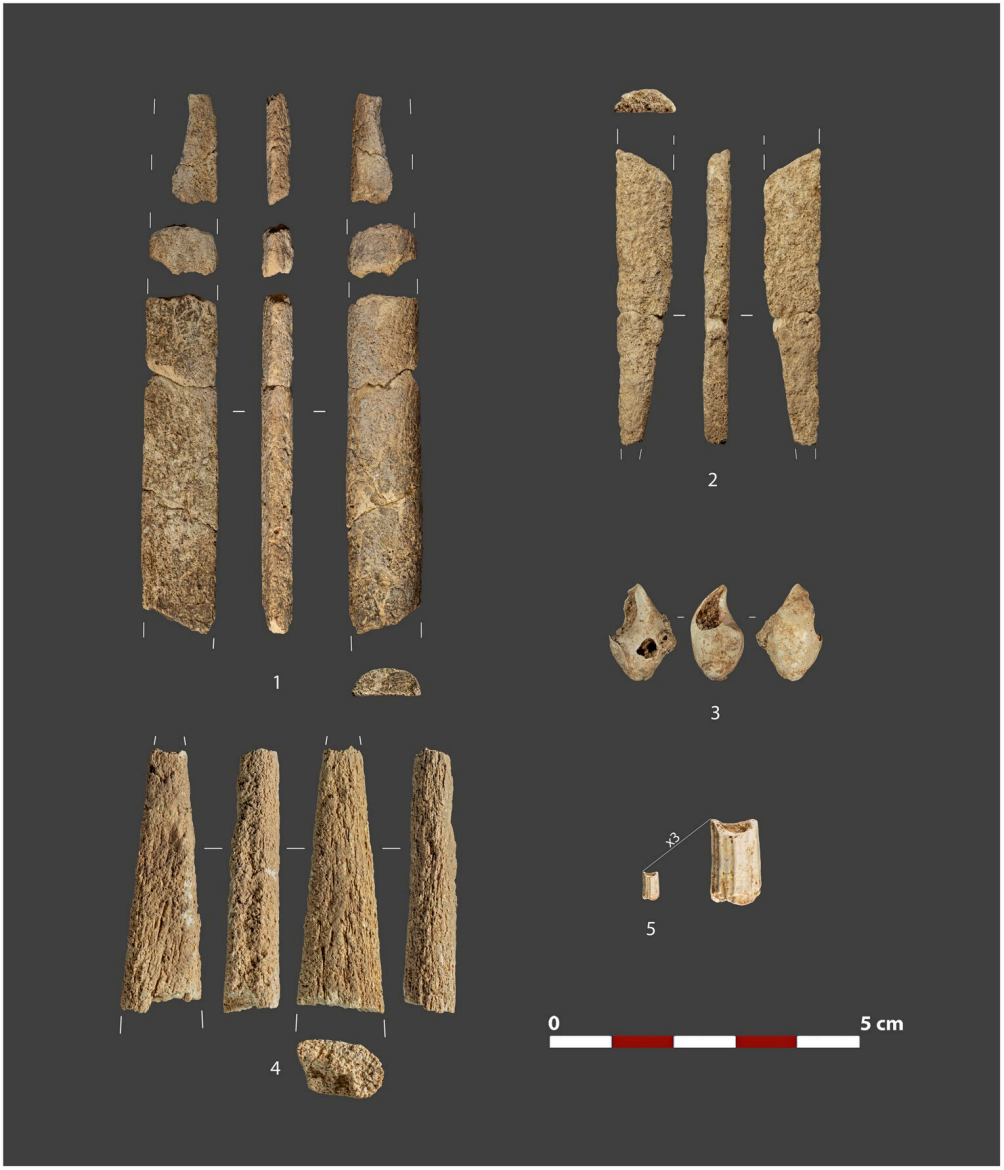
Selection of osseous artifacts and personal ornaments collected at Level 1 of Charco Verde II. 1 & 2: Fragments of baguettes demi-ronde (half-round rods). 3: Perforated mollusk. 4: Fragment of sagaie (antler point). 5: Dentalium.

The lithic assemblage of Level 1 is composed of 2,909 items classified as knapping products, although 1,469 are flake fragments and chips <1 cm. 928 products (31,90%) are complete, while 1,981 (68,10%) present fractures. Among surface alterations, white patinas or dehydration are recorded in 441 flint pieces (15.16% of the total assemblage and 15.48% of the flint collection), while rounding is absent and pseudo-retouches are only found marginally. Thermal alterations, including color change, potlids (oval-shaped scalar fractures), and internal cracking (interpreted as the result of fire exposure) are found in 496 flint artifacts (17.05% of the total assemblage and 17.42% of the flint collection).

Exploited raw materials are mostly focused on flint (97.90% of the lithic products), part of which has a potential catchment source just 30 km away from the site, in the Concha outcrop, as suggested by the macroscopic inspection of lithic products. Flint pieces are followed by those knapped on quartzite and hyaline quartz or rock crystal, but they are comparatively scarce (Table 6). Leaving aside microdébitage and chunks, which sum up to more than half of the lithic products, raw blanks are the most represented technological category, followed by retouched artifacts, cortical flakes and preparation products, burin spalls, core maintenance by-products and, lastly, cores (Table 6). According to these data (see also S2 Appendix), the exploitation stage (phase II) of the *chaîne opératoire* is the most represented (84.17%, not considering chunks and microdébitage), followed by the consumption and abandonment stage (phase III) (12.01%) and the initialization stage (phase I), which is poorly recorded (3.82%).

**Table 6:** Quantitative distribution of lithic technological categories and raw materials recorded in Level I of Charco Verde II rock shelter (see S2 Appendix for a complete classification).

Technological categories/Raw materials	Flint	Quartzite	Hyaline quartz	Total	%	% (+ microdébitage)
Cores	9	0	0	9	0.63	0.31
Cortical flakes & preparation products	31	24	0	55	3.82	1.89
Core maintenance by-products	13	0	0	13	0.90	0.45
Knapping waste (chunks & microdébitage)	1,466	2	6	1,474	0.35	50.67
Raw blanks	1,159	27	2	1,188	82.50	40.84
Retouched blanks	150	0	0	150	10.42	5.16
Tool maintenance by-products (burin spalls)	20	0	0	20	1.39	0.69
**Total (excluding microdébitage)**	1,382	53	5	1,440	100	*
**% (excluding microdébitage)**	95.97	3.68	0.35	100	*	*
**Total (+ microdébitage)**	2,848	53	8	2,909	*	100
**% (+ microdébitage)**	97.90	1.82	0.28	100	*	*

Concerning knapping methods and objectives, the raw blanks are dominated by bladelets (41.50%), followed by blades (20.29%) small flakes (20.03%) and flakes (18.185), most of them produced on flint (97.56%), especially in the case of the laminar productions, with only 1 blade on hyaline quartz and 1 bladelet produced on a fine-grained quartzite (Table 7). All recorded cores are on flint, and they include two unidirectional blade cores and seven bladelet cores, most of them of prismatic shapes (Fig 14.8 & 14.11), but also two pyramidal shaped cores and one burin-core. Therefore, data recorded thus far shows that knapping aims were focused on the production of bladelets and blades, including large-sized ones (up to 9 cm long) (Fig 14.5 & 14.7), although the latter probably were not produced on-site, as no large cores or core by-products have been found to date. Although the studied assemblage is scarce, as the excavation area is still limited (in fact, the assemblage is relatively large in relation to the excavated area), obtained results points to a relatively fragmented operational sequence. The low presence of cortical flakes, preparation products, and cores (Table 6 & S2 Appendix) suggest that lithic products were mostly brought into the rock shelter after initial preparation and first knapping of cores were developed elsewhere. However, these results must be considered subject to change as fieldwork continues in the coming years.

**Table 7:** Quantitative distribution of lithic blanks (including fragmented products) and raw materials recorded in Level I of Charco Verde II rock shelter (see S2 Appendix for a complete classification).

Raw blank categories/Raw materials	Flint	Quartzite	Hyaline quartz	Total	%
Blades	240	0	1	241	20.29
Bladelets	492	1	0	493	41.50
Flakes	189	26	1	216	18.18
Small flakes (2–1 cm)	238	0	0	238	20.03
**Total**	1159	27	2	1188	100
**%**	97.56	2.27	0.17	100	*

As for retouched artifacts, the inventory is strongly dominated by backed bladelets, which account for the 65.33% of the tools, and thus can be considered one of the main objectives of the lithic production. They are followed by burins, endscrapers, notches, denticulates, continuously retouched pieces, backed blades, truncated pieces and other items showing undiagnostic typological features (Table 8 and Fig 14). Most of the tools were produced on bladelets (65.33%) and blades (27.33%), while only 7.33% of them were produced on flake blanks.

**Table 8:** Typological groups and tool types identified in the lithic assemblage of Level I of Charco Verde II. Numbers in parentheses refer to the Sonneville-Bordes and Perrot type-list [101].

Typological group	Tool type	Blank	Raw Material	#	%
**Endscrapers**				**12**	8
	Simple endscraper (1)	Blade	Flint	7	
	Endscraper on retouched blade (5)	Blade	Flint	4	
	Carinated endscraper (11)	Blade	Flint	1	
**Burins**				**14**	9.33
	Straight dihedral burin (27)	Blade	Flint	3	
	Canted dihedral burin (28)	Blade	Flint	7	
	Canted dihedral burin (28)	Flake	Flint	1	
	Angle burin on break (30)	Blade	Flint	3	
**Backed blades**				**2**	1.33
	Partially backed blade (59)	Blade	Flint	2	
**Truncated pieces**				**2**	1.33
	Obliquely truncated piece (61)	Blade	Flint	1	
	Convex truncated piece (63)	Blade	Flint	1	
**Continuously retouched pieces**				**3**	2
	Piece with continuous retouch on one edge (65)	Blade	Flint	3	
**Notches and denticulates**				**13**	8.67
	Notch (74)	Blade	Flint	5	
	Notch (74)	Flake	Flint	2	
	Denticulate (75)	Blade	Flint	4	
	Denticulate (75)	Flake	Flint	2	
**Backed bladelets**				**98**	65.33
	Backed bladelet (85)	Bladelet	Flint	89	
	Pointed backed bladelet (85b)	Bladelet	Flint	2	
	Denticulated backed bladelet (87)	Bladelet	Flint	7	
**Others**		Flake	Flint	**6**	4
**TOTAL**				**150**	100

Concerning the osseous tools, we have documented several fragments of sagaie points on antlers, mostly from deer, including pointed and flattened morphologies, with at least one example of lengthwise grooving. Other artifacts include at least two fragmented *baguettes demi-ronde* (half-round rods), which, as all other bone tools and faunal remains, are heavily affected by surface alterations, and hence do not allow the identification of any potential engraved decoration. Finally, after wet-screening of sediments we have identified four perforated shell beads, one of them potentially bearing ochre remains, and two fragments of *dentalium* (Fig 15).

The presence of fire at Level 1 is attested by the relevant number of charcoal pieces recovered (Table 2), as well as by the high number of bone and lithic remains affected by fire (17.5% of the total assemblage in the case of lithics). Furthermore, the presence of ash-gray sediments at different areas, including a circular concentration potentially associated to quartzite and limestone cobbles in square 1B (Fig 13), suggest the existence of fireplaces or combustion features at the site.

Overall, the recent discovery of the Charco Verde II site has shown a rich and relevant archeological record, which demonstrates the recurrent occupation of the rock shelter by humans spanning, more or less intermittently, ∼5300–6300 years, throughout the Magdalenian sequence. The high number of backed bladelets, burins and endscrapers, together with the presence of osseous artifacts, and especially shell beads and two *baguettes demi-rondes*, enable us to assign Level 1 to the Middle or Upper Magdalenian, based on techno-typological grounds when comparing with the archeological record both at the Iberian [5] and regional level [102, 123]. This cultural attribution is well justified even without considering obtained radiocarbon results in the range of 15.1 and 16.6 ka cal BP. Yet, these dates confirm such attribution, as they span the timeframe of the Middle and initial Upper Magdalenian as defined in Iberia [5, 124]. Since archeological assemblages excavated at levels 2 to 5 are still very scarce, no precise cultural assignment is yet possible for them. However, considering the radiocarbon dates obtained for Level 5, ranging from 20.8 to 21.4 ka cal BP, it is reasonable to suggest that the base of the sequence corresponds to either the early stages of the Magdalenian or the last phases of the Solutrean, as there are well-dated assemblages in Iberia assigned to both technocomplexes in that time range [5, 125–127]. However, given the dates of 21.0–22.8 ka cal BP obtained for the Archaic Magdalenian assemblages of the Gato 2 site, in the nearby Jalón River valley (see below), our working hypothesis is that Level 5 of Charco Verde II attest for a Lower or Archaic Magdalenian occupation. If confirmed, this site would join La Peña de Estebanvela [102] and Buendía [128] as rock shelters bearing long sequences of recurrent Magdalenian occupations in inland Iberia. The relevant feature of Charco Verde II is, however, that the human presence at this site starts during the Last Glacial Maximum, a phase which is absent in the mentioned sequences. In fact, the only evidence attesting for Magdalenian human occupation during the LGM in the whole Iberian interior is found at the mentioned level of Gato 2 [123], while a radiocarbon date on bone of 20.3–20.8 ka cal BP obtained at Portalón de Cueva Mayor (Atapuerca), for which a direct association to human presence is not warranted [129], cannot yet be considered.

## Discussion and final remarks

### The sequence of human occupation at Charco Verde II and the settlement of inland Iberia during the cold periods of the Last Glacial

The Charco Verde II archeological site is located in an interior and upland region of the Iberian Peninsula, at the southwestern foothills of the Iberian System range, at the drainage divide between the Ebro and Tagus basins. It has shown a sequence of recurrent Magdalenian occupations spanning the GS-2 period, including the cold and arid phases of the LGM and HS 1. Its discovery presents relevant scientific implications despite fieldwork at the site is still in a preliminary stage. These implications revolve around two main circumstances:

1. Although the Magdalenian is, by far, the most represented technocomplex of the Upper Paleolithic in inland Iberia [15, 28, 123, 130], well-dated sites bearing sequences of recurrent human occupations are still few, and its initial phases are poorly known (Table 9). In this regard, Charco Verde II is an unprecedented discovery.
2. Although some Magdalenian occurrences in inland Iberia are related to cold spells during GS-2 and GS-1 (see below), only one site, Gato 2, has been previously related to the LGM (Table 9). The Charco Verde II sequence not only unequivocally starts at the height of the LGM, but it has shown paleoenvironmental data confirming that human occupations registered at the rock shelter during this time frame occurred within harsh environmental conditions. Furthermore, the biogeographic setting of the site, in a region which nowadays faces one of the coldest climates in the Iberian Peninsula, makes this fact even more relevant for discussing human-climate-environment interactions during the Last Glacial.

**Table 9:** Sequence of archeological levels with chronometric dates for the Magdalenian of inland Iberia. ^(1)^ These levels show uncertainties on the relation between archeological assemblages and obtained dates. ^(2)^ These levels show uncertainties on their assignation to a specific Magdalenian techno-cultural phase.

Techno-cultural pase	Archeological levels	ka cal BP	References
**Final Magdalenian**	Fariseu—4	11.1–12.6_AMS_	[137]
	Peña del Diablo—1	12.5–12.9_14C_	[150]
	Estebanvela—I & II	12.5–13.6_AMS_	[102]
**Upper Magdalenian**	Quinta da Barca Sul—3	11.5–12.7_TL_	[137]
	Foz do Medal– 1055^(1)^	11.4–13.2_OSL_	[36]
	Estebanvela—III & IV	13.7–15.2_AMS_	[102]
	Verdelpino—VA & VB^(1) (2)^	14.7–17.6_14C_	[135]
	Charco Verde II—1^(2)^	15.1–16.6_AMS_	This study
**Middle Magdalenian**	Peña de Santana—3^(1) (2)^	16.0–17.8_AMS_	[147]
	El Monte—I & II^(2)^	16.2–18.2_AMS_	[133]
	Estebanvela—VI	17.1–17.8_AMS_	[102]
**Lower Magdalenian**	Buendía—1W to 33 C^(2)^	15.7–18.6_AMS_	[128]
	Vergara—d	16–7–17.3_AMS_	[41]
	Alexandre—IIIb	18.3–18.8_14C_	[41]
	Ojo Guareña–Galería Huellas^(1) (2)^	18.3–19.4_14C_	[143]
	Portalón Cueva Mayor—10P1^(1) (2)^	20.3–20.8_AMS_	[129]
	Charco Verde II—5^(2)^	20.8–21.4_AMS_	This study
**Archaic Magdalenian**	Gato 2–2	21.1–22.9_AMS_	[149]

**Fig 16:**
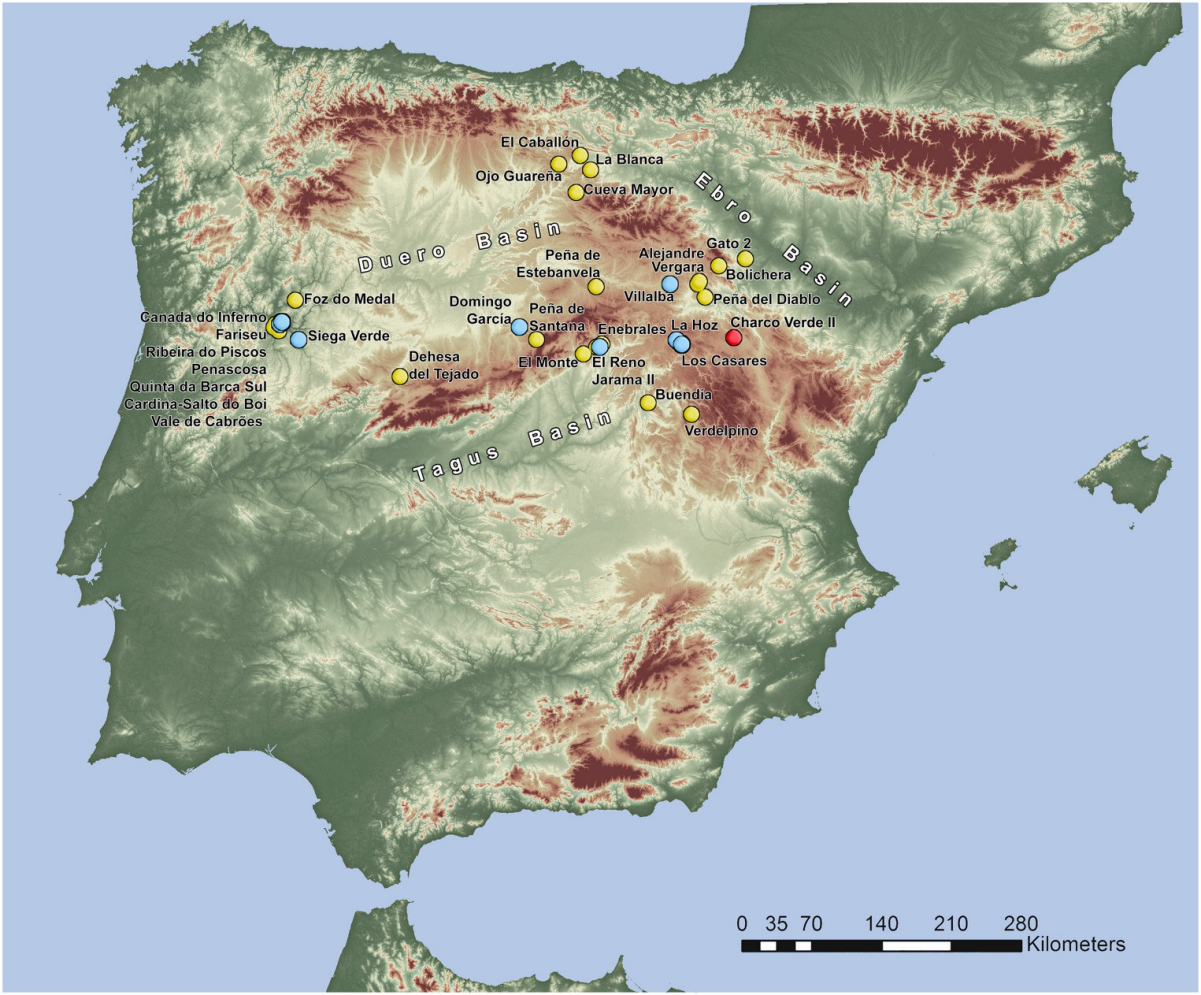
Sites with archeological deposits (yellow) or rock/mobile art (blue) assigned to the Magdalenian in inland Iberia. Charco Verde II is highlighted in red. Map generated with ArcGIS (ArcMap 10.3.1.) using ASTER Global Digital Elevation Model V0032019, distributed by NASA EOSDIS Land Processes DAAC, (10.5067/ASTER/ASTGTM.003) (free for use: https://lpdaac.usgs.gov/products/astgtmv003/).

**Fig 17:**
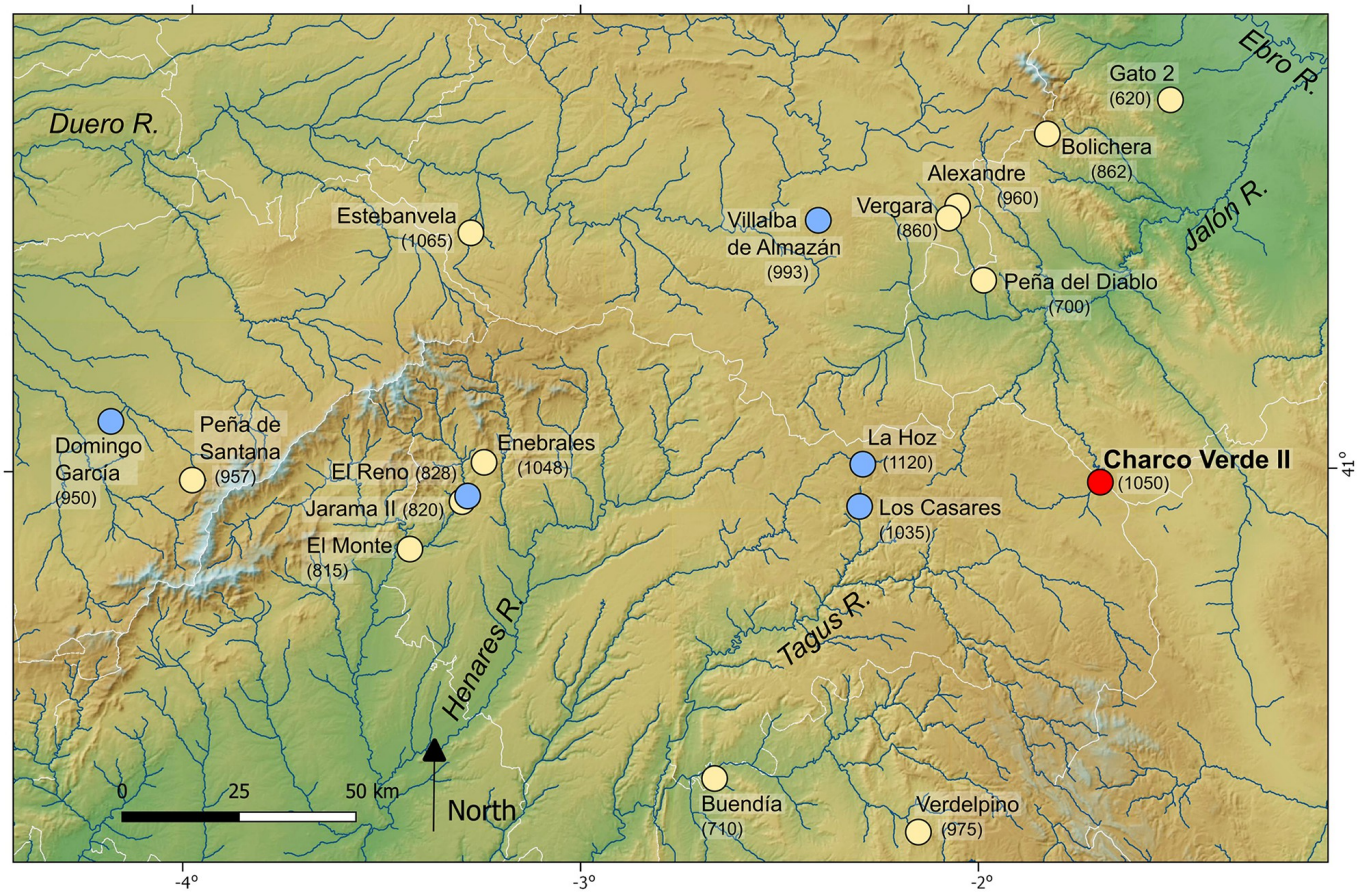
Location and altitude of Magdalenian sites in the right bank of the Middle Ebro basin and the eastern part of the northern and southern Mesetas (see Fig 16 for a larger view in the Iberian Peninsula). Map generated with QGIS 3.4 using base cartographic data obtained from Centro Nacional de Información Geográfica, modified from PNOA_MDT200_ETRS89_HU30 csn.es (free for use: https://centrodedescargas.cnig.es/CentroDescargas/index.jsp) and fluvial network from Ministerio para la Transición Ecológica y el Reto Demográfico (free for use: https://www.miteco.gob.es/es/cartografia-y-sig/ide/descargas/agua/red-hidrografica.aspx).

When considering the Magdalenian sequence of Charco Verde II in its regional context, the first aspect to note is that no Magdalenian or Upper Paleolithic settlement is known in its immediate surroundings (Figs 16 & 17). The moorlands of Sigüenza and Molina de Aragón, traditionally considered a hostile region for hunter-gatherers due to its altitude and harsh environments, had only shown human presence during the Last Glacial at the classic Paleolithic cave art sites of Los Casares and La Hoz, where Magdalenian-style engravings and paintings are known [43, 53]. To the west, still in the Guadalajara province, in the Central System southern foothills there are two sites assigned to the Magdalenian, none of which haven shown radiometric dates: the Jarama II cave [131] and Los Enebrales rock shelter [132]. Following the Jarama valley, just 45 km southwest we find El Monte rock shelter (Madrid province), where two archeological levels containing blades, bone tools and personal ornaments have been radiocarbon dated between 16.2 and 18.2 ka cal BP [133]. The last two sites in the southern Meseta are found in the Buendía and Verdelpino rock shelters (Cuenca province). In Buendía there is a long stratigraphic sequence bearing Magdalenian assemblages, radiocarbon dated by 18 assays to 15.7–18.6 ka cal BP (i.e. in the time range of the Middle and mostly Lower Magdalenian) [128, 134]. Yet, in Verdelpino only conventional and unprecise ^14^C dates are available, although the Magdalenian nature of the lithic assemblages is sound [135, 136] (Table 9).

In the northern Meseta (Fig 16), sites are also few, and some of them are located in the borders of the plateau, such as the important cluster of the Côa River Valley, in the Portuguese Douro basin. Here, Magdalenian assemblages are found in the sites of Fariseu, Quinta da Barca Sul and Cardina-Salto do Boi [35, 137], and Magdalenian-style open-air engravings have been described at Canada do Inferno, Ribera do Piscos, Penascosa and Vale do Cabrões among some others [138]. Also in Portugal, in the nearby site of Foz do Medal (Upper Sabor River valley), there is a Magdalenian layer rich in mobile art [36, 139]. Still in the Duero basin, but in the Spanish side, the open-air rock art sites of Siega Verde [140] and, to a lesser extent, Arroyo de las Almas [141], show rock art depictions assigned to Magdalenian times. Also in Salamanca, the open-air site of Dehesa del Tejado showed a large assemblage rich in backed bladelets and burins, which has been attributed to the Magdalenian despite no chronometric dates are available [142]. In the northern border of the plateau, to the problematic site of Portalón de Cueva Mayor (see above), it could be added the nearby sites of La Blanca and El Caballón (Burgos), for which only old news about potential Magdalenian occupations are known, however [22]. Finally, a charcoal dated to 18.3–19.4 ka cal BP, potentially associated to human footprints at Ojo Guareña (Galería de las Huellas), attest for the transit of this cave during Magdalenian times [143, 144].

Still in the northern Meseta, but on the northern foothills of the Central System range, and hence closer to Charco Verde (Fig 16), we find the long sequence recorded at La Peña de Estebanvela rock shelter, in the Segovia province [102, 130]. This site has shown, to date, the most complete sequence for understanding the Magdalenian human settlement in inland Iberia. This is composed of six archeological levels ranging from 12.5 to 17.1 ka cal BP, and rich in artifacts, faunal remains, mobile art and combustion features [102, 130, 145]. Also in the Segovia province, the open-air site of Domingo García presents rock art engravings assigned to the Magdalenian [146]. Finally, the recently discovered site of La Peña de Santana rock shelter (Segovia) has shown Magdalenian assemblages in three archeological levels, one of which has been radiocarbon dated to 16.0–17.8 ka cal BP [147]. To the east, in the Soria province, the plaque of Villalba de Almazán, although an isolated surface finding devoid of archeological context, shows engraved depictions of sound Magdalenian style [148].

The Magdalenian occupations of the Jalón and Henar River valleys, connecting the Middle Ebro basin with the Meseta, are closely related to the environment where Charco Verde is located. In fact, they are the closest Magdalenian sites to Charco Verde besides Los Casares and La Hoz (Fig 16). Put together, the sites known in the Jalón and Henar valleys cover a large part of the Magdalenian sequence (Table 9). Thus, level 2 of the Gato 2 rock shelter (Zaragoza province), radiocarbon dated to 21.1–22.9 ka cal BP, has been assigned to the Archaic Magdalenian, the Vergara and Alexandre rock shelters (Soria province) haven shown Lower Magdalenian assemblages between 16.7 and 18.8 ka cal BP, and Level 1 of Peña del Diablo (Zaragoza province) has been defined as a Final Magdalenian assemblage dated to 12.5–12.9 ka cal BP [41, 123, 149]. Another nearby site is found in Bolichera cave (Zaragoza province), for which radiometric dates are not available, but showed a unilaterally barbed harpoon, thus suggesting an Upper Magdalenian chronology [150].

In this context, the discovery of a sequence of Magdalenian occupations at the Charco Verde II rock shelter, located at the drainage divide between the Ebro and Tagus basins, could be understood as part of a large cultural network connecting the northeastern fringe of the southern Meseta with the right bank of the Middle Ebro valley during the Magdalenian. The cultural relations between both regions have been already claimed by P. Utrilla and colleagues [41, 123, 149] for different periods of the Upper Paleolithic, especially through the natural corridor of the Jalón River valley. In this regard, it must be considered that most of these sites are located at high altitudes in mountainous and moorlands landscapes. In fact, Charco Verde II, with an altitude of 1050 m asl and a surrounding relief that reaches ∼1200 m in its immediate vicinity, is one of the highest Magdalenian sites in the whole Iberian hinterland, with only Estebanvela (1065 m), and La Hoz (1120) above it. The Ebro sites are in slightly lower landscapes, but still high and mountainous (Fig 17). Therefore, the increasing archeological record of this territory (Fig 17) shows the recurrent human occupation of an interior and upland area of Spain which, as the whole Iberian hinterland, had been traditionally considered inhospitable for the settlement of humans during the cold periods of the Last Glacial due its harsh environmental and climate conditions. In this regard, it is worth considering that Charco Verde II is located in one of the coldest regions of modern-day Spain, as discussed above.

As shown by radiocarbon data, the sequence of human occupation at Charco Verde II include periods during the LGM (Level 5) and HS 1 (Level 1). Studies based on paleoenvironmental proxies [8, 151], paleoglaciers [152] and sedimentological data [153] at the regional and Iberian level, as well as off-shore pollen records [154, 155] and paleoclimate modelling [23, 25], all show a general trend of cold climates and arid environments during both phases, and especially during HS 1. Paleoenvironmental data gathered at Charco Verde II, and especially the pollen record, demonstrate that this was in fact the case for part of the human occupation sequence, as levels 5 to 3 are dominated by steppic landscapes associated to arid and very cold conditions. However, levels 1 and 2, roughly associated to HS 1, although still shows a vegetation cover typical of continentalized climates, suggest a more temperate environmental and climatic setting. Human occupations registered at these levels, corresponding to the Middle-Upper Magdalenian, were thus developed during not-so-cold and arid conditions, probably matching one of the short warm spells within the HS 1. Anyhow, the recurrent presence of hunter-gatherer groups at the site during both harsh and mild environmental conditions, points to the high adaptability and resilience of these groups, which occupied the high and continental regions of Iberia not only during warm episodes, but also during the cold and arid peaks of the LGM. Therefore, contrary to the classic idea positing that humans avoided this type of ecological contexts during the Last Glacial, the Charco Verde II site demonstrates that Late Pleistocene hunter-gatherers were capable of adapting to a wide variety of landscapes and environments, including upland and very cold regions [156–160]. Naturally, there are still some questions to be solved, such as the seasonality of the occupations, as environmental conditions would have been different in summer and winter. However, the faunal assemblages recovered to date do not yet allow for conducting studies on seasonality, including cementochronology, mesowear, and microwear on herbivore teeth. Once these assemblages are enlarged in the coming years, these analyses will hopefully contribute to this issue.

Anyhow, Charco Verde II is not the only site of the Iberian interior which have shown human occupations during the cold phases of GS-2. This is also the case of levels V and VI of Estebanvela, which most probably include periods in the timeframe of HS 1 [102]. These levels show Middle Magdalenian assemblages associated to micromammals and archaeobotanical data confirming the existence of cold environments and open landscapes at that time [102, 145]. In Buendía, cold and dry conditions are also recorded by different paleoenvironmental proxies both at the bottom and top of the Magdalenian sequence, being most of it in the range of HS 1 [128]. In La Peña de Santana, dates for level 3 are also coincident with GS-2.1a and HS 1 [147]. Finally, among the sites in the Ebro Valley, Gato 2 corresponds to the LGM, Vergara and Alexandre include layers related to GS-2 and HS 1, while Peña del Diablo is related to GS-1 (roughly equivalent to the Younger Dryas) [123]. Although in the case of Buendía their excavators hypothesize that human occupations correspond to temporal incursions of groups based in the Mediterranean region [128, 134], and Alexandre, Vergara and Peña del Diablo environments are favored by the presence of thermal waters in the surroundings [123], the general pattern is sound. The growing concentration of Magdalenian occupations during periods of cold and dry conditions in these upland regions, being Charco Verde II the most recent addition, reflects a marked contrast with the classic idea limiting the Last Glacial settlement of the Iberian interior to punctuated occupations or ephemeral visits during interstadial periods or warm spells. As it has been recently demonstrated for previous phases of the Upper Paleolithic, population dynamics in the Iberian interior were far less limited by the environmental conditions imposed by the cold and arid periods of MIS 2, as shown by the recurrent occupations recorded at the Peña Capón rock shelter during HS 2 [32]. The site of Charco Verde II, located at a height of 1050 meters in one of the coldest populated regions of modern-day Spain, and showing several occupation periods during two of the coldest phases of the Last Glacial, has revealed as an important source for moving forward on this topic. Thus, it is increasingly clear that population dynamics and patterns of land use in the Iberian Peninsula during the Upper Paleolithic include a far more complex, dense, and recurrent human settlement of the interior lands than previously believed, to be found even in the most unsuspected regions. The process of building a new paradigmatic picture to overcome the classic idea of the Iberian interior as a ‘no-man’s land’ during the coldest phases of the Last Glacial, still in the making as fieldwork increases, has in Charco Verde II a new stepping-stone.

## Supporting information

S1 Appendix Bayesian modeling of radiocarbon dates from Charco Verde II sequence. (PDF)

S2 Appendix Technological study of lithic assemblages recorded at Charco Verde II—Level 1. (XLSX)
