# Supplementary material for: Human occupations of upland and cold environments in inland Spain during the Last Glacial Maximum and Heinrich Stadial 1: The new Magdalenian sequence of Charco Verde II
Source: PLoS One. 2023 Oct 4;18(10):e0291516. doi: 10.1371/journal.pone.0291516 (PMC10550185; doi:10.1371/journal.pone.0291516)
Supplement: S1 Appendix — (PDF) [file pone.0291516.s001.pdf]

Human occupations of upland and cold environments in inland Spain during the Last Glacial Maximum and Heinrich Stadial 1: the new Magdalenian sequence of Charco Verde II

J. Aragoncillo-del Río [1,2], J.J. Alcolea-González [3], L. Luque [3], S. Castillo-Jiménez [3], G. Jiménez-Gisbert [3], J.A. López-Sáez [4], J.M. Maíllo-Fernández [5,6], M. Ruiz-Alonso [4], I. Triguero [3], J. Yravedra [7], M. Alcaraz-Castaño [3,]*

(1) Molina-Alto Tajo UNESCO Global Geopark, Molina de Aragón (Guadalajara), Spain.
(2) Escuela Internacional de Doctorado de la UNED (EIDUNED), Spain
(3) Area of Prehistory (Department of History and Philosophy), University of Alcalá, Alcalá de Henares, Spain
(4) Environmental Archeology Research Group, Institute of History, CCHS CSIC, Madrid, Spain
(5) Institute of Evolution in Africa (IDEA), University of Alcalá, Madrid, Spain
(6) Department of Prehistory and Archaeology, Universidad Nacional de Educación a Distancia (UNED), Madrid, Spain.
(7) Department of Prehistory, Complutense University, Madrid, Spain.

* Corresponding author: manuel.alcaraz@uah.es (MA-C)

Supporting Information

S1 Appendix. Bayesian modeling

Table S1*. Detailed results of the Bayesian Model built with the radiocarbon dates obtained for the Charco Verde II sequence. Modeled calibrated ages and Boundaries with the 68.2% and 95.4% probability ranges. Calculated with OxCal 4.4 [1], using a General t-type Outlier Model [2] and IntCal20 [3].*

**Charco Verde II**	**Unmodeled (cal BP)**				**Modeled (cal BP)**				**Indices**<br>**Amodel=101.5**<br>**Aoverall=101.4**		
	**68.2% probability range**		**95.4% probability range**		**68.2% probability range**		**95.4% probability range**				
	**from**	**to**	**from**	**to**	**from**	**to**	**from**	**to**	**A**	**P**	**C**
End Boundary					15436	13900	15582	10340			97.9
OxA-41038 (13152,33)	15842	15698	15924	15644	15844	15696	15930	15640	100.9	96.4	99.8
COL7029.1.1 (13616,62)	16550	16332	16660	16244	16550	16332	16674	16234	100.6	96.1	99.8
COL7028.1.1 (12822,61)	15426	15190	15548	15124	15444	15208	15564	15130	99.2	96.2	99.8
↑Level 1 Phase											
OxA-42475 (17412,52)	21050	20894	21210	20848	21052	20892	21204	20844	101.6	96.4	99.9
OxA-X-3179-16 (17554,83)	21350	21054	21430	20930	21314	21020	21420	20922	101.2	96.1	99.4
↑Level 5 Phase											
Start Boundary					22576	21064	26172	20952			97.2
Charco Verde II Sequence											

S1 Text*. CQL Codes for Bayesian analysis*

```
Options()
 {
  Resolution=20;
 };
 Plot()
 {
  Outlier_Model("General",T(5),U(0,4),"t");
  Sequence("Charco Verde II")
  {
   Boundary("Start");
   Phase("Level 5")
```

```
  {
   R_Date("OxA-X-3179-16", 17554, 83)
   {
    Outlier(0.05);
   };
   R_Date("OxA-42475", 17412, 52)
   {
    Outlier(0.05);
   };
  };
  Phase("Level 1")
  {
   R_Date("COL7028.1.1", 12822, 61)
   {
    Outlier(0.05);
   };
   R_Date("COL7029.1.1", 13616, 62)
   {
    Outlier(0.05);
   };
   R_Date("OxA-41038", 13152, 33)
   {
    Outlier(0.05);
   };
  };
  Boundary("End");
 };
};
```

Supplementary references

1. Bronk Ramsey C. Bayesian analysis of radiocarbon dates. Radiocarbon. 2009;51: 337–360. doi:10.1017/s0033822200033865

2. Bronk Ramsey C. Dealing with outliers and offsets in radiocarbon dating. Radiocarbon. 2009;51: 1023–1045. doi:10.1017/s0033822200034093

3. Reimer PJ, Austin WEN, Bard E, Bayliss A, Blackwell PG, Bronk Ramsey C, et al. The IntCal20 Northern hemisphere radiocarbon age calibration curve (0–55 cal kBP). Radiocarbon. 2020;62: 725–757. doi:10.1017/rdc.2020.41
